# Condensation properties of stress granules and processing bodies are compromised in myotonic dystrophy type 1

**DOI:** 10.1242/dmm.049294

**Published:** 2022-08-02

**Authors:** Selma Gulyurtlu, Monika S. Magon, Patrick Guest, Panagiotis P. Papavasiliou, Kim D. Morrison, Alan R. Prescott, Judith E. Sleeman

**Affiliations:** 1 Biomolecular Sciences Research Complex, School of Biology, University of St Andrews, North Haugh, St Andrews, Fife KY16 9ST, UK; 2 Division of Cell Signalling and Immunology, School of Life Science, University of Dundee, Dundee DD1 5EH, UK

**Keywords:** LLPS, Myotonic dystrophy type 1, P-bodies, Stress granules, Trinucleotide repeats

## Abstract

RNA regulation in mammalian cells requires complex physical compartmentalisation, using structures thought to be formed by liquid-liquid phase separation. Disruption of these structures is implicated in numerous degenerative diseases. Myotonic dystrophy type 1 (DM1) is a multi-systemic trinucleotide repeat disorder resulting from an expansion of nucleotides CTG (CTGexp) in the DNA encoding DM1 protein kinase (DMPK). The cellular hallmark of DM1 is the formation of nuclear foci that contain expanded *DMPK* RNA (CUGexp) (with thymine instead of uracil). We report here the deregulation of stress granules (SGs) and processing bodies (P-bodies), two cytoplasmic structures key for mRNA regulation, in cell culture models of DM1. Alterations to the rates of formation and dispersal of SGs suggest an altered ability of cells to respond to stress associated with DM1, while changes to the structure and dynamics of SGs and P-bodies suggest that a widespread alteration to the biophysical properties of cellular structures is a consequence of the presence of CUGexp RNA.

## Abstract (precis)

**Summary:** Validation of an inducible model of myotonic dystrophy type 1 that shows altered cellular stress responses. These involve phase-separated cellular structures also implicated in other degenerative conditions.

## INTRODUCTION

Myotonic dystrophy type 1 (DM1) is an autosomal dominant, multi-systemic disease (reviewed by Pettersson et al., 2015) with an estimated global prevalence of 1:20,000 (Theadom et al., 2014). Its symptoms are variable and can include myotonia, muscular atrophy, cardiac defects, cataracts and insulin resistance (Pettersson et al., 2015). DM1 is caused by an expansion of nucleotides cytosine, thymine and guanine (CTGexp) in the DM1 protein kinase (*DMPK*) gene. Transcription of mutant *DMPK* results in RNA transcripts containing a (CUG)*_n_* repeat expansion (CUGexp) – with uracil (U) instead of thymine (T) – in their 3′ untranslated region (3′UTR), which then form an elongated metastable stem-loop structure (Pettersson et al. 2015). These RNA transcripts accumulate to form nuclear foci (CUGexp foci) and recruit RNA-binding proteins (RBPs), notably muscleblind-like protein 1 (MBNL1), which interacts dynamically with the CUGexp foci, showing rapid exchange with MBNL1 (Coleman et al., 2014; Ho et al., 2005). Similarities between DM1 and DM2 – the latter resulting from a similar repeat expansion within an unrelated gene (Liquori et al., 2001) – suggest that the nuclear CUGexp foci are the source of DM1 pathology. The mechanisms by which CUGexp foci result in cellular damage and the broad range of DM1 symptoms are not understood but are proposed to include disturbance of the normal cellular functions of MBNL1.

MBNL1 is the most abundant RNA metabolism regulator within the muscleblind-like protein family and expressed in most tissues (Konieczny et al., 2014). The protein has a key role as an alternative splicing regulator, acting antagonistically with a second splicing regulator, i.e. CUGBP Elav-like family member 1 (CELF1, also known as and hereafter referred to as CUGBP1) (Junghans, 2009; Mahadevan et al., 2006). The splicing activity of CUGBP1 is increased in DM1 and thought to be linked to accumulation of MBNL1 in CUGexp foci. Splicing alterations attributed to disturbance of the balance between active MBNL1 and CUGBP1 have been documented in DM1. For example, whereas MBNL1 inhibits exon-5 inclusion in mRNA of cardiac troponin T (TNNT2), CUGBP1 promotes it (Wang et al., 2007), and inclusion of exon-11 in the insulin receptor (INSR) is promoted by MBNL1 and inhibited by CUGBP1 (Savkur et al., 2001). However, both MBNL1 and CUGBP1 have additional diverse functions in gene regulation. MBNL1 is also implicated in RNA transport and stability, and in miRNA processing (Konieczny et al., 2014; Osborne et al., 2009; Rau et al., 2011; Wang et al., 2012). CUGBP1 is involved in translation, stability and decay of mRNA (Masuda et al., 2012; Teplova et al., 2010).

Cellular RNA metabolism is complex, and its physical organisation within the cell involves the formation of RNA-rich bodies within the nucleus and cytoplasm. In the nucleus, these include Cajal bodies, nuclear speckles and paraspeckles, whereas – in the cytoplasm – structures including processing bodies (P-bodies) and stress granules (SGs) play roles in RNA stability. These are all non-membranous, highly dynamic structures and there is evidence that at least some of them are formed by liquid-liquid phase separation (LLPS) (Banani et al., 2016; Jain et al., 2016; Kroschwald et al., 2015; Protter and Parker, 2016; Stanĕk and Fox, 2017). LLPS requires a high concentration of a molecule or a mixture of molecules, forming a network of weak multivalent interactions resulting in a separate phase. This allows structures to exhibit liquid-like properties, such as having spherical, droplet-like morphology resulting from reduced surface tension, and demonstrating fusion, fission and reversible disruption (Courchaine et al., 2016; Sawyer et al., 2019). Phase-separated structures normally allow rapid exchange of components, which distinguishes them from the more-solid protein aggregates (Markmiller et al., 2018; Stanĕk and Fox, 2017) that are implicated in pathologies, such as amyotrophic lateral sclerosis (ALS) and frontotemporal dementia (FTD) (Jain et al., 2016; Li et al., 2013; Markmiller et al., 2018; Ramaswami et al., 2013).

In mammalian cells, as in cells of other species, P-bodies and SGs (Jain et al., 2016; Protter and Parker, 2016) are cytoplasmic structures proposed to form by phase separation dependant on mRNA-protein interactions, with key roles in cellular signalling and the stress response (Anderson et al., 2015; Protter and Parker, 2016). They interact with each other both physically and functionally (Anderson et al., 2015; Protter and Parker, 2016; Shah et al., 2016), and share a number of protein components, although the precise relationship between the two structures is unclear. P-bodies contain the RNA decay machinery, including the decapping enzymes DCP1A, and DCP2, and decapping activators, such as EDC4 and DDX6 (Anderson et al., 2015). P-bodies appear to be built on a scaffold of mRNA (Banani et al., 2016; Ditlev et al., 2018) and have been specifically implicated in mRNA degradation, surveillance, translation repression and RNA-mediated gene silencing (Anderson et al., 2015; Protter and Parker, 2016). SGs derive from mRNAs stalled during translation initiation, typically resulting from forms of cellular stress, such as oxidative stress, heat shock, hypoxia, starvation and viral infection (Anderson et al., 2015; Protter and Parker, 2016; Roy and Rajyaguru, 2018). In contrast to the smaller P-bodies, mammalian SGs appear to contain a combination of liquid and more-solid phases, where different areas within the SG exhibit varying levels of dynamic exchange of their components (Jain et al., 2016; Protter and Parker, 2016). In yeast, SGs have a more-solid structure (Ditlev et al., 2018; Jain et al., 2016; Kroschwald et al., 2015). Upon removal of stress, SGs disassemble (Hardy et al., 2017; Mateju et al., 2017; Protter and Parker, 2016; Turakhiya et al., 2018) and messenger ribonucleoproteins (mRNPs) that, during the stress response, had been sequestered within them return to active translation (Lee and Seydoux, 2019). Aberrant persistent SGs, which are predicted to be less dynamic than canonical SGs, have been implicated in neurodegenerative diseases, such as ALS and FTD (Jain et al., 2016; Markmiller et al., 2018; Wolozin and Ivanov, 2019; Zhang et al., 2018), and have been proposed to result from increased liquid-to-solid phase transitions within the SGs, with autophagy implicated in their clearance (Mateju et al., 2017).

Increased cellular stress, resulting from the presence of expanded *DMPK* RNA (CUGexp) RNA foci, has been suggested to be a pathological effector in DM1 (Huichalaf et al., 2010; Ihara et al., 1995; Kumar et al., 2014; Toscano et al., 2005; Usuki and Ishiura, 1998; Usuki et al., 2000). Both MBNL1 and CUGBP1 have been identified in SGs of mammalian cells (Fujimura et al., 2008; Onishi et al., 2008), and increased numbers of SGs have been reported in DM1 myoblasts as compared to SG numbers in control myoblasts grown under normal conditions (Huichalaf et al., 2010). Furthermore, a defect in SG formation in response to treatment with sodium arsenite (NaAsO_2_) has been reported in DM1 patient-derived fibroblasts expressing exogenous MyoD to mimic a myoblast phenotype, suggesting impaired ability of DM1 cells to respond to stress (Ravel-Chapuis et al., 2016).Here, we identified both MBNL1 and CUGBP1 as components of P-bodies as well as SGs in human lens epithelial cells (HLECs) and in a novel inducible cell model of DM1. Analysis of the dynamic behaviour of MBNL1 and CUGBP1 in live cells further implicates CUGexp RNA and MBNL1 as regulators of the formation and turnover of cytoplasmic SGs, suggesting a mechanism by which the presence of CUGexp RNA and disruption of the subcellular distribution of MBNL1 results in deregulation of SGs and, by extension, the fine control of cytoplasmic RNA metabolism in DM1.

## RESULTS

The hallmark cellular defect in DM1 is the presence of CUGexp RNA foci formed by mutant DMPK transcripts in the cell nucleus, within which the multi-functional RBP MBNL1 accumulates. While some DMPK mRNA is clearly exported to the cytoplasm and translated (Gudde et al., 2017), the global effect of the presence of CUGexp RNA and the formation of nuclear CUGexp foci on cellular RNA metabolism is not understood. Lens cataract is the most prevalent symptom in DM1 (Romeo, 2012; Smith and Gutmann, 2016), and we have previously determined that the CUGexp foci in HLECs derived from DM1 patients contain only 0.2-0.5% of cellular MBNL1 (Coleman et al., 2014), making sequestration of MBNL1 within the foci an unlikely explanation for DM1-associated cataract. Transparency of the lens is achieved by the differentiation of a stem cell pool within the lens epithelium, during which transcription is shut down, and all cellular organelles and inclusions are lost (Dahm and Prescott, 2002; Gribbon et al., 2002). Lens formation relies on forms of autophagy, i.e. the same process that is responsible for clearance of SGs under certain conditions (Hardy et al., 2017; Mateju et al., 2017; Protter and Parker, 2016; Turakhiya et al., 2018). Together, evidence for alteration of SGs in DM1 and our previous observation that MBNL1 localises to SGs following transcriptional arrest in HLECs (Coleman et al., 2014) led us to investigate the presence of the DM1-associated proteins MBNL1 and CUGBP1 in cytoplasmic structures involved in RNA metabolism.

### MBNL1 and CUGBP1 colocalise within P-bodies in HLECs

**Fig. 1.**
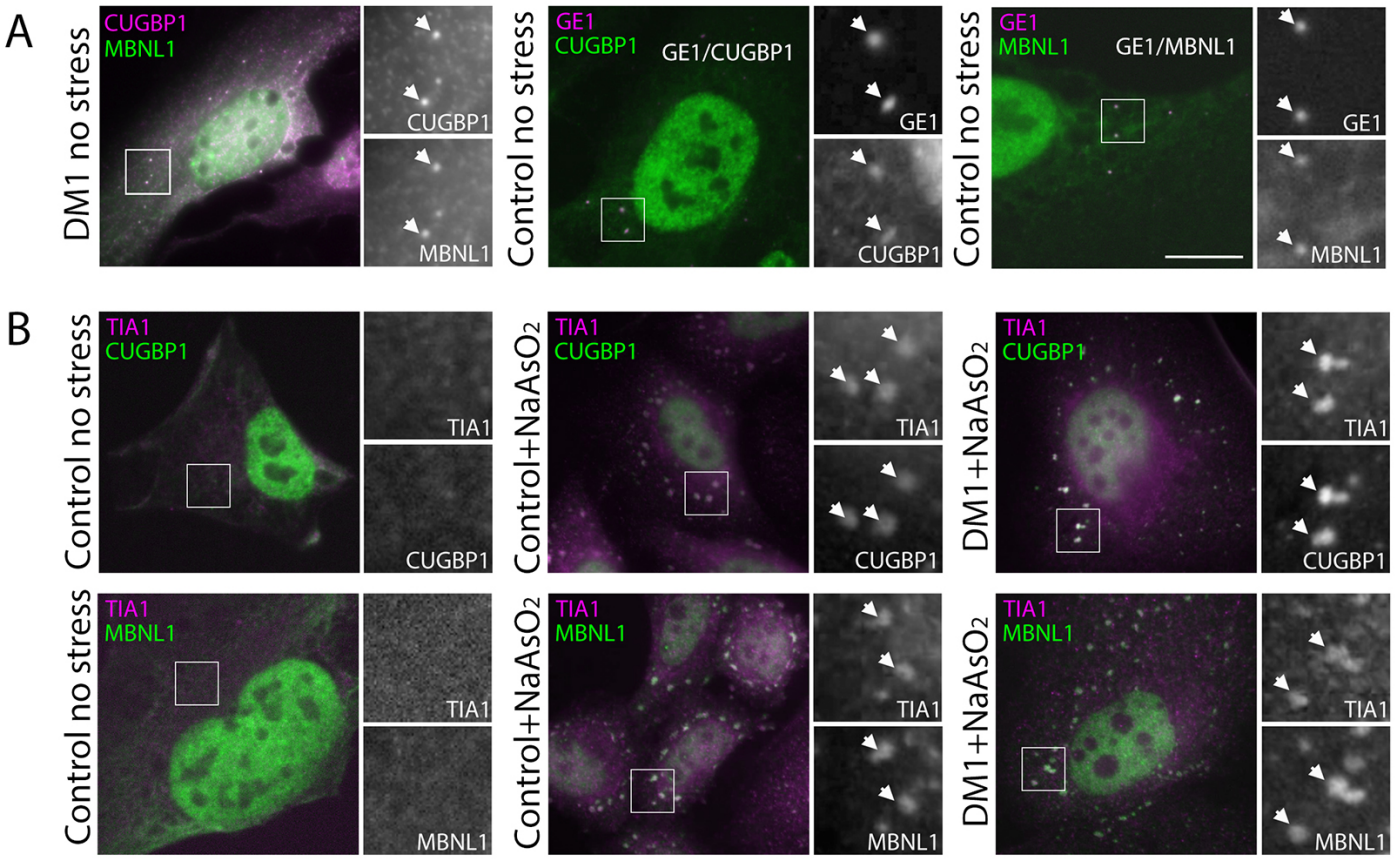
**MBNL1 and CUGBP1 both localise to P-bodies within HLECs and to SGs following treatment with NaAsO_2_.** (A) Endogenous MBNL1 (left panel, green on overlay) and CUGBP1 (left panel, magenta on overlay) colocalise in punctate cytoplasmic structures (arrows) despite showing little colocalisation in the nucleus (see also Fig. S1). Counterstaining with antibodies against the P-body marker GE1 (centre and right panels, magenta on overlay) demonstrates that both CUGBP1 (centre panel, green on overlay) and MBNL1 (right panel, green on overlay) localise to P-bodies (arrows) in DM1 and control cell lines (see also Fig. S1). (B) SGs detected with antibodies against TIA1 (magenta on overlay) are not seen in unstressed cells (left panels). Following treatment with 0.5 mM NaAsO_2_ for 45 min, control cells (centre panels) and DM1cells (right panels) show clear cytoplasmic SGs (arrows) identified by antibodies against TIA1 (magenta on overlays) containing both CUGBP1 (top row green on overlays) and MBNL1 (bottom row, green on overlays; see also Fig. S1). Boxed areas are shown magnified at the right of each panel; scale bar: 7 µm.

Our initial examination of the subcellular distribution of endogenous CUGBP1 and MBNL1 in HLECs (four patient-derived cell lines and two age-matched control cell lines) revealed a striking contrast between their distributions in the nucleus, where MBNL1 localises to CUGexp foci and CUGBP1 does not, and the cytoplasm, where the two proteins colocalise in discrete punctate structures (Fig. 1A and Fig. S1). Further analysis demonstrated these structures to be P-bodies, identified by using the P-body marker GE1 (Fig. 1A). Whereas accumulation of CUGBP1 has been reported in P-bodies (Yu et al., 2013), MBNL1 has not previously been reported as a P-body component. Our observation of both these proteins in P-bodies points to close links between the cytoplasmic functions of MBNL1 and CUGBP1.

### MBNL1 and CUGBP1 both accumulate within SGs of HLECs following treatment with NaAsO_2_

MBNL1 and CUGBP1 have both been reported to accumulate within SGs (Fujimura et al., 2008; Kedersha and Anderson, 2009) in different cell types, using varied strategies to induce cellular stress. Furthermore, myoblasts from DM1 patients have been reported to display SGs under normal growth conditions, suggesting a higher basal level of stress compared to controls (Huichalaf et al., 2010). Although we saw no evidence of SGs in the DM1 HLEC lines under normal growth conditions (Fig. 1B), the close relationship between P-bodies – where both proteins colocalise – and SGs led us to investigate the effects of stress on the cytoplasmic distribution of MBNL1 and CUGBP1. We used NaAsO_2_ to induce SG formation in HLECs. NaAsO_2_ induces SGs through oxidative stress by activating the metabolic-stress-sensing protein kinase EIF2AK1 (Aulas et al., 2017), making it a suitable stress effector to use in the context of DM1 HLECs. Using TIA1 as a marker for SGs, we showed that SGs form readily in HLECs in response to NaAsO_2_, and that MBNL1 and CUGBP1 both accumulate in SGs (Fig. 1B).

### SGs show no delay in formation but disperse more quickly in DM1 HLECs compared to SGs in control cells

**Fig. 2.**
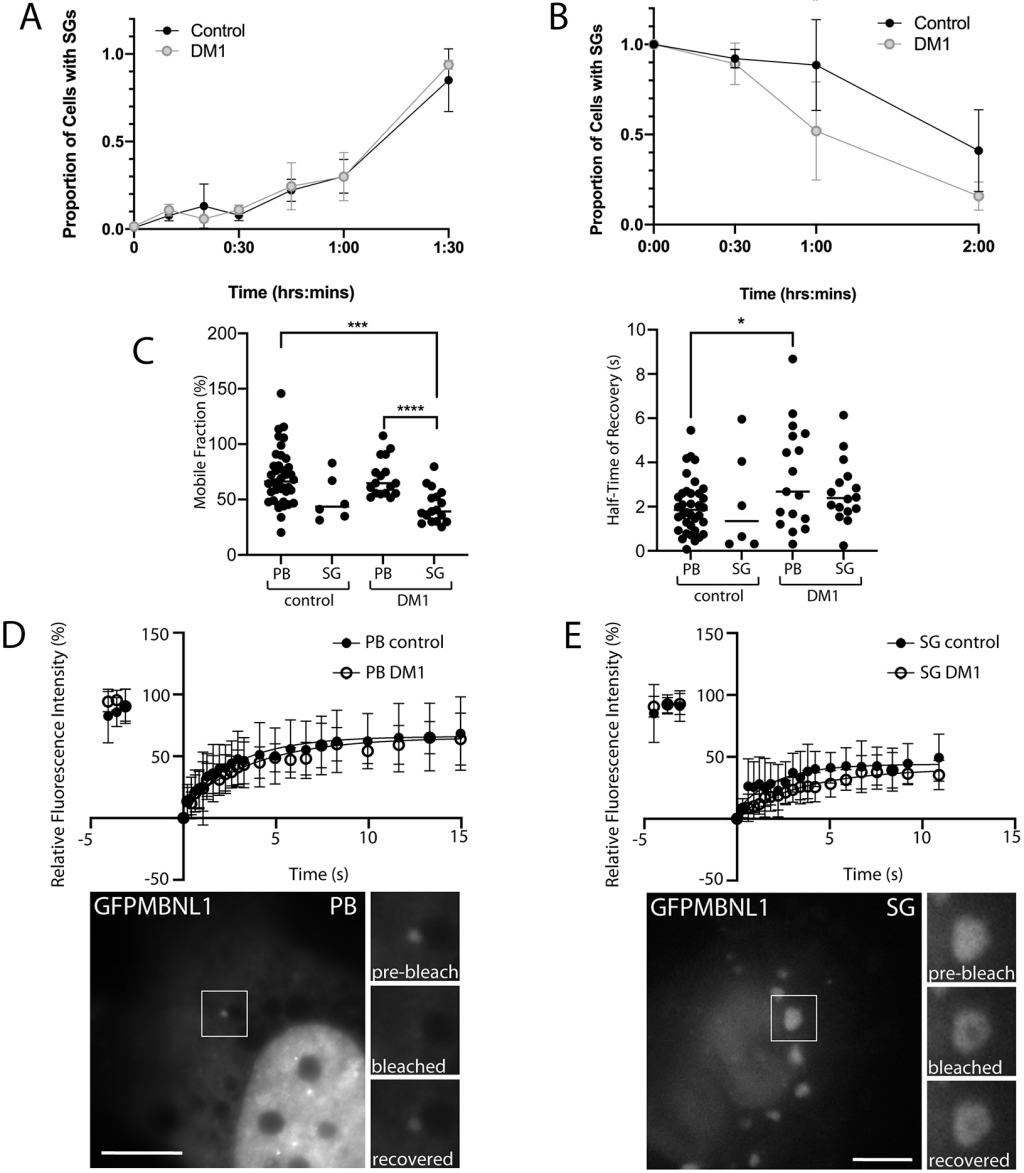
**Kinetics analysis of SGs and P-bodies reveals increased rates of SG loss during stress recovery, and slightly altered MBNL1 dynamics in DM1 compared to control cells.** (A) SG formation. Control and DM1 HLECs treated with 0.25 mM NaAsO_2_ were followed over 1.5 h during which the proportion of cells containing SGs was detected with antibody against endogenous TIA1. No significant differences were observed between DM1 and control cells. (B) SG recovery after pre-treatment with NaAsO_2_. Cells were pre-treated with NaAsO_2_ (0.5 mM for 90 minutes), washed thoroughly, and the proportion of cells containing SGs was detected over 2 h of recovery with antibodies against endogenous TIA1 and MBNL1. Compared with control cells, significantly fewer DM1 cells retain SGs at the 1 h time point. *P*<0.05 one-way ANOVA (*n*=50 cells per cell line per time-point); data shown are pooled from two independent experiments, obtained from three DM1 cell lines and two control cell lines (see Fig. S2 for individual data from each cell line). (C) Mobile fraction (left) and half-time of fluorescence recovery (right) of GFPMBNL1 within P-bodies (PB) and SGs (SG) in control and DM1 HLECs. *P<0.05; ***P<0.001; ****P<0.0001 Kruskal–Wallis. (D,E) Plotted is the relative fluorescence recovery of GFP-tagged MBNL1 (GFPMBNL1) in P-bodies (PB; panel D) and SGs (SG; panel E) over 15 s, following photobleaching, using a 488 nm laser pulse for 2.5 s. Closed circles indicate P-bodies or SGs in control cells [HLECs from CCat1 and CCat3 (see ‘Cell lines and cell culture’ in Materials and Methods), *n*=39 total for P-bodies and *n*=6 for SGs]; open circles indicate P-bodies or SGs in DM1 cells [HLECs from DMCat1, DMCat2 and DMCat4 (see ‘Cell lines and cell culture’ in Materials and Methods), *n*=17 total for P-bodies and *n*=17 for SGs]. All data are displayed as the mean±s.d. Representative images before photobleaching are shown below, magnifications of the boxed areas are shown on the right of each image, before and immediately after photobleaching, and following recovery. Scale bars: 7 µm.

Having shown that SGs are not present in DM1 HLECs under normal growth conditions – suggesting that any increase in basal stress levels is insufficient to drive SG formation – and that SGs containing MBNL1 and CUGBP1 are readily formed in response to NaAsO_2_, we next sought to investigate the kinetics of SG formation and SG loss in DM1 HLECs compared to control cells. When fixed after differing lengths of treatment with NaAsO_2_, neither DM1 HLECs nor control HLECs showed any delay in the formation of SGs detected using endogenous TIA1 (Fig. 2A). In contrast, DM1 HLECs that were pre-treated with NaAsO_2_ to induce SGs and then allowed to recover showed a more rapid loss of SGs detected using endogenous TIA1 and MBNL1, compared to control HLECs (Fig. 2B). This suggests that DM1 cells differ from control cells in their ability to recover from stress.

### MBNL1 dynamics are unaffected within SGs in DM1 HLECs but altered in P-bodies

We next used fluorescence recovery after photobleaching (FRAP) to examine the dynamics of MBNL1 in SGs and P-bodies, by using transiently expressed GFP-tagged MBNL1 (hereafter referred to as GFPMBNL1), previously validated to behave similarly to endogenous MBNL1 in these cell lines (Coleman et al., 2014). Previous studies have shown that, in mammalian cells, these two related structures are both highly dynamic, displaying properties of liquid droplets (Kroschwald et al., 2015). Persistent SGs have long been associated with degenerative conditions, notably ALS (Li et al., 2013; Wolozin and Ivanov, 2019), but the dynamics of MBNL1 within SGs and P-bodies in the context of DM1 has not been examined.

To analyse the dynamics of SGs, HLECs transiently expressing GFPMBNL1 were treated for 45 min with 0.5 mM NaAsO_2_ to induce SGs. To analyse the dynamics of P-bodies, HLECs were transiently transfected to express both GFPMBNL1 and mCherry-Dcp1a, a marker for P-bodies, to enable their unambiguous identification. A diffraction-limited spot of laser excitation was used to bleach GFPMBNL1 in SGs or P-bodies and the subsequent recovery fitted to a one-phase exponential recovery model (Fig. 2C-E and Fig. S3). This modelling (Fig. 2C) showed no statistically significant changes in the mobile fraction or half-time of recovery of GFPMBNL1 in SGs in DM1 cells compared to controls, suggesting that SGs, once formed, are not defective in DM1 cells in terms of MBNL1 dynamics. The mobile fraction of GFPMBNL1 within P-bodies in both DM1 and control cells was significantly larger than that for SGs in DM1 cells (Fig. 2C), suggesting that GFPMBNL1 has a more-dynamic relationship with P-bodies than with SGs, although the small number of data points for SGs in control cells prevents a strong conclusion from being drawn (69.6±17.2% for P-bodies in DM1, 70.1±25.2% for P-bodies in control, 43.9±15.2% for SGs in DM1, 50.6±20.2% for SGs in control). A significant increase in the half-time of recovery for GFPMBNL1 in P-bodies was seen for DM1 cells compared to controls, indicating an altered relationship of MBNL1 with P-bodies associated with DM1 (3.4±2.3 s for DM1, 2.0±1.2 s for control).

### Simultaneous expression of a *DMPK* minigene and GFPMBNL1 models DM1 cells with CUGexp foci detected in living cells

Patient-derived HLECs are a highly relevant cell model in which to study the cellular pathology of DM1. However, the use of patient cell lines comes with the key drawback that the lines are of diverse genetic origins, making disease onset and severity as well as length of CUG repeats different between them. To assess the effect of CUGexp RNA on SG structure and dynamics directly, we generated an inducible HeLa cell model for DM1. The stable HeLa cell lines comprising this model contain a bi-directional tetracycline-responsive promoter that drives the simultaneous expression of GFPMBNL1 and a *DMPK* minigene. To mimic the DM1 phenotype, this *DMPK* minigene contains an interrupted 960-nucleotide-long CTG repeat (CTG960), yielding model cell line HeLa_CTG_960__GFPMBNL1. However, as the disease only manifests in the presence of >50 repeats (Lee et al., 2012), the *DMPK* minigene in the matched control cell line, i.e. HeLa_CTG_12__GFPMBNL1, contains only12 CTG repeats (CTG12).

**Fig. 3.**
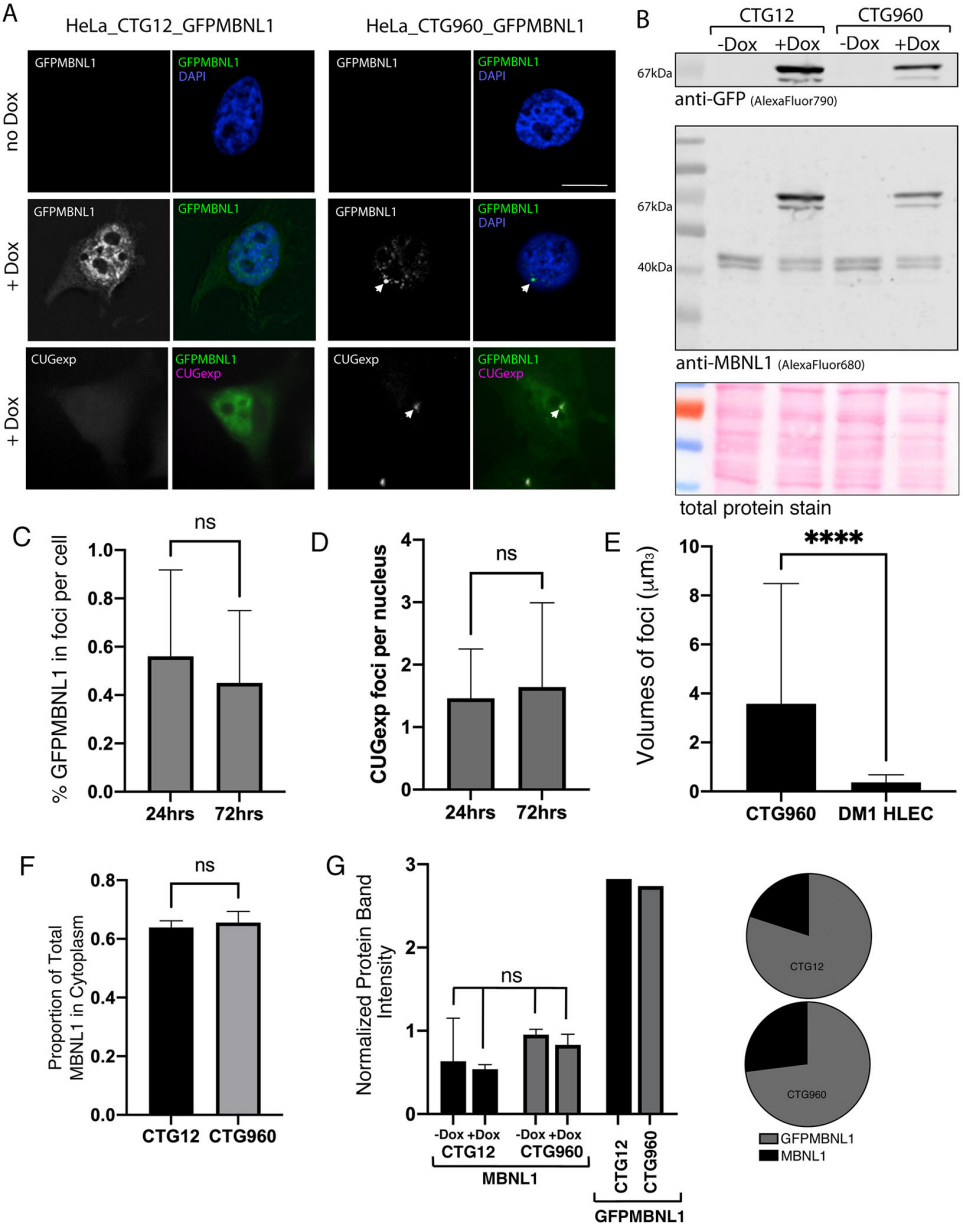
**Simultaneous expression of a *DMPK* minigene and GFPMBNL1 models DM1.** (A) HeLa cells containing a *DMPK* minigene with 12 (left) or 960 (right) CTG trinucleotide repeats and GFP-tagged MBNL1 (GFPMBNL1), under a bi-directional tetracycline responsive promoter. Cells grown in the absence of doxycycline (−Dox) (top row) show no expression of GFPMBNL1 in the cytoplasm or nucleus (counterstained with DAPI, blue on overlay). Cells induced with 1 µg/ml Dox (+Dox) for 48 h show clear expression of GFPMBNL1 (middle row, green on overlay), forming distinct nuclear foci (arrows) in the CTG960 line and the characteristic localisation of GFPMBNL1 to the nucleus and cytoplasm in the CTG12 line. RNA FISH using a probe against the CUGexp RNA (bottom row, magenta on overlay) confirms that the GFPMBNL1 nuclear foci are genuine CUGexp foci in the CTG960 line (right) and that no CUGexp RNA is detected in the CTG12 line (left). Scale bar: 10 µm. (B) Immunoblotting of PAGE-separated whole-cell lysates using antibodies against GFP (AlexaFluor790; top) and MBNL1 (AlexaFluor680; centre) confirmed successful expression of GFPMBNL1 (67 KDa) in both cell lines, i.e. CTG12 (left) and CTG960 (right), following treatment with Dox for 48 h – but not in uninduced cells (−Dox). All samples show the characteristic double-band just above the 40 kDa marker for endogenous MBNL1 (centre). Ponceau S staining of total protein (bottom) was used as loading control. (C) The percentage of total cellular GFPMBNL1 localised to foci is small (<1%) and does not change between 24 h and 72 h of induction (*n*=48 cells at 24 h and *n*=68 cells at 72 h, pooled from two independent experiments; unpaired *t*-test). (D) The number of CUGexp foci per nucleus is small (mean 1.7 and 2.4 after 24 h and 72 h, respectively; *n*=84 cells at 24 h and *n*=117 cells at 72 h, pooled from two independent experiments; Mann–Whitney *U* test). (E) CUGexp foci are larger in HeLa cells than in DM1 HLECs (*n*=419 cells for HeLa, *n*=86 cells for HLEC; ****P<0.0001 Mann–Whitney *U* test. (F) The proportion of total cellular GFPMBNL1 in the cytoplasm is similar in CTG12 and CTG960 cell lines after 72 h of induction (*n*=10 cells per line). (G) Expression levels of endogenous MBNL1 are not significantly different in CTG12 and CTG960 cells, whether induced with Dox or not (ANOVA test, mean of two biological repeats adjusted for gel loading by using tubulin. Expression levels of GFPMBNL1 and the ratio of GFPMBNL1 to endogenous MBNL1 as shown in panel B are similar in CTG12 and CTG960 cells, adjusted for the number of cells expressing GFPMBNL1 in each cell line. All data are displayed as the mean+s.d.

Fluorescence microscopy and immunoblotting confirmed that no detectable GFPMBNL1 was expressed in the absence of the tetracycline equivalent doxycycline (Dox), whereas addition of Dox resulted in expression of GFPMBNL1 in both cell lines (GFPMBNL1 in Fig. 3A and 67 kDa band in Fig. 3B). In the control cell line HeLa_CTG_12__GFPMBNL1>95% of cells expressed GFPMBNL1, showing the characteristic subcellular distribution of endogenous MBNL1 in the nucleus and, more weakly, in the cytoplasm. In the DM1 model cell line HeLa_CTG_960__GFPMBNL1>70% of cells expressed GFPMBNL. Clear nuclear accumulations of GFPMBNL1 were seen in addition to the usual distribution in >95% of expressing cells (Fig. 3A and Fig. S4). These were confirmed as CUGexp foci by using RNA fluorescence *in situ* hybridisation (FISH) with a Cy3-CAG_10_ probe against the trinucleotide repeats (CUGexp in Fig. 3A), allowing nuclear GFPMBNL1 accumulation to be used as a marker for CUGexp foci in subsequent experiments. There are typically several CUGexp foci per nucleus, also seen in HLECs derived from DM1 patients (Fig. 3D see also Figs 5, 8, Figs S1, S4, Movies 2 and 4 and Coleman et al., 2014). The partition of GFPMBNL1 between cytoplasm and nucleus was not affected by the presence of CUGexp foci (Fig. 3F). Comparison of the signals for endogenous MBNL1 and GFPMBNL1, detected with antibody against MBNL1 by immunoblotting, revealed moderate overexpression of GFPMBNL1 in both cell lines (Fig. 3B,G), with <1% of GFPMBNL1 accumulating in CUGexp foci (Fig. 3C). The number of foci did not change significantly between 24 h and 72 h of induction (Fig. 3D) but the foci were significantly larger than those previously characterised in DM1 HLECs (Fig. 3E; Coleman et al., 2014).

### In DM1 HeLa cell models, MBNL1 and CUGBP1 show subtle changes in colocalisation within P-bodies and sub-regions of SGs, associated with the presence of CUGexp foci

**Fig. 4.**
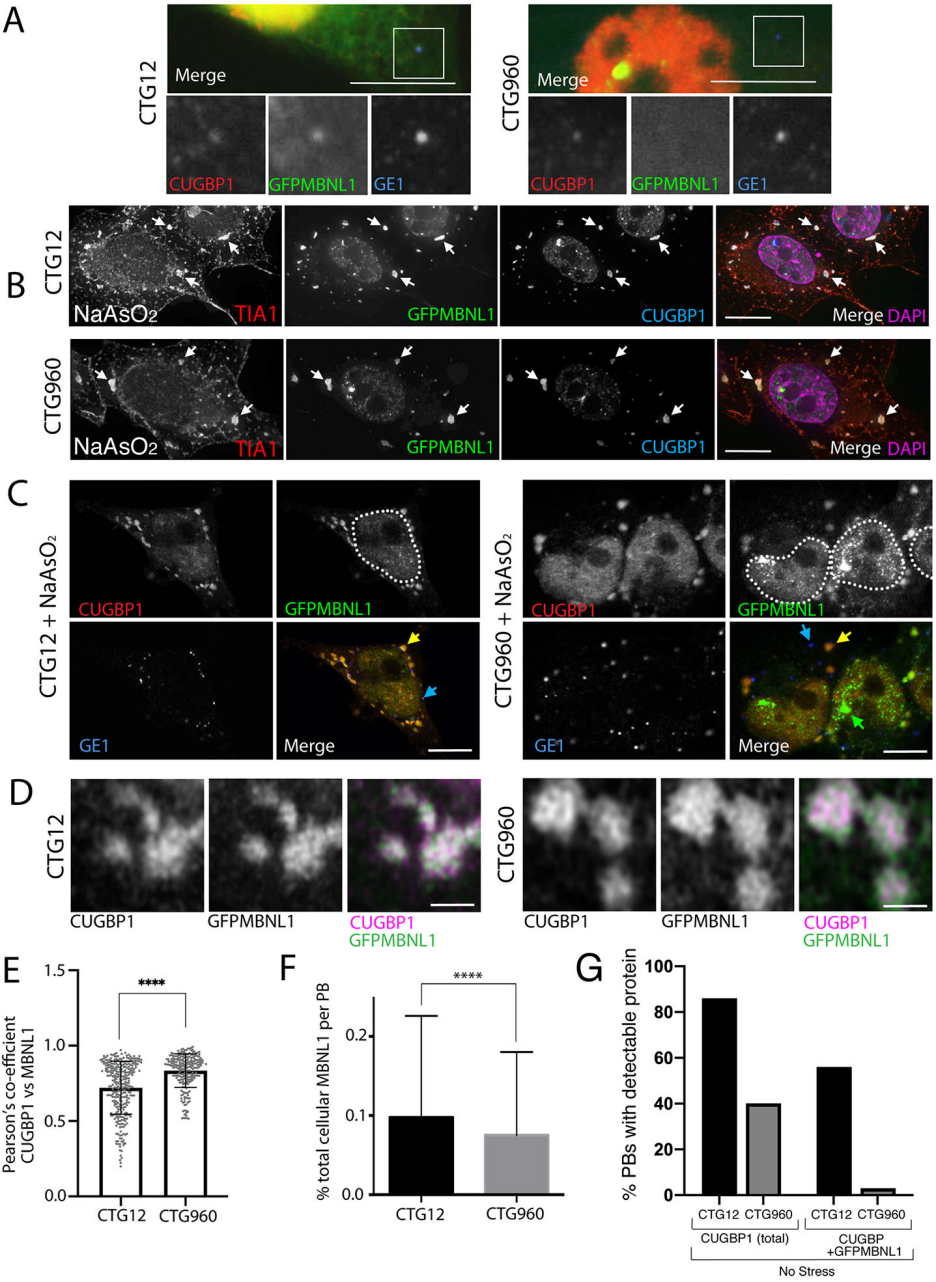
**MBNL1 and CUGBP1 colocalise in P-bodies and sub-regions of SGs in HeLa models of DM1. (**A) Unstressed HeLa_CTG_12__GFPMBNL1 (CTG12, left) and HeLa_CTG_960__GFPMBNL1 (CTG960, right) stained for CUGBP1 (red), GFP-tagged MBNL1 (GFPMBNL1; green) and GE1 (blue). For CTG12 cells, the merged image (top) shows colocalization of GE1 with CUGBP1 and GFPMBNL1 in cytoplasmic P-bodies. For CTG960 cells, the merged image (top), shows colocalization of GE1 with CUGBP1 in P-bodies, but GFPMBNL1 (green) is not detectable. Magnified images of the boxed P-bodies are shown below. Scale bars: 10 µm. Red and green signals in these images have been adjusted to visualise the cytoplasmic P-bodies, resulting in saturation of the nuclear signal. (B) CTG12 (top) and CTG960 (bottom) cells after treatment with NaAsO_2_, showing SGs (arrows) containing TIA1 (red in merged image), GFPMBNL1 (green in merged image) and CUGBP1 (blue in merged image). Scale bars: 10 µm. (C) CTG12 (left) and CTG960 (right) cells treated with NaAsO_2_. SGs (yellow arrows) show colocalisation of CUGBP1 (red in merged images) and GFPMBNL1 (green in merged images). Blue arrows highlight large number of P-bodies (detected with GE1, blue in merged images) also present in both cell lines. Nuclear foci of GFPMBNL1 (green in merged image) are only present in CTG960 cells (green arrow). Dotted lines show the approximate outlines of nuclei by using GFPMBNL1 signal. Scale bars: 10 µm. (D) Super-resolution airyscan images of SGs showing non-uniform distribution of CUGBP1 (magenta in merged images) and GFPMBNL1 (green in merged images) in CTG12 (left) and CTG960 (right) cells. Scale bars: 1 µm. (E) Graph of Pearson correlation coefficient of colocalisation between CUGBP1 and GFPMBNL1 in SGs of CTG12 and CTG960 cells (****P<0.00001; *n*=204 SGs and *n*=229 SGs in CTG960 and CTG12 cells, respectively, from three independent experiments; unpaired *t*-test). (F) Analysis of the amount of GFPMBNL1 in the P-bodies of CTG12 and CTG960 cells that had been treated with NaAsO_2_. The percentage of GFPMBNL1 in P-bodies of CTG_960_ cells (*n*=670 P-bodies from 53 cells) was reduced compared with that in P-bodies of CTG_12_ cells (*n*=924 P-bodies from 34 cells); ****P<0.0001 unpaired *t*-test). (G) Unstressed CTG_960_ and CTG_12_ cells were induced with doxycycline for 72 h. P-bodies (in %) that contain CUGBP1 or CUGBP1 plus MBNL1 are reduced in CTG_960_ cells compared with levels in CTG_12_ cells (*n*=100 cells per cell line per experiment). All data are displayed as the mean±s.d. (see also Fig. S5).

In unstressed Dox-induced HeLa_CTG_12__GFPMBNL1 and HeLa_CTG_960__GFPMBNL1 cells, P-bodies were less numerous and less distinct than in HLECs (Fig. 4A, Fig. S5). Many of the P-bodies present in the control HeLa_CTG_12__GFPMBNL1 cell line accumulated small amounts of both GFPMBNL1 and CUGBP1, with 86% of P-bodies containing CUGBP1 and 56% of P-bodies both proteins (*n*=57 cells). In the DM1 model line HeLa_CTG_960__GFPMBNL1, very few P-bodies contained detectable levels of GFPMBNL1, while fewer P-bodies contained CUGBP1 compared to the control line, i.e. 40% of P-bodies contained CUGBP1 but only 3% contained both proteins (*n*=53 cells) (Fig. 4G). This suggests that P-body components, particularly MBNL1, were altered by the presence of the CTG960 expansion. Following treatment with NaAsO_2_ to induce cellular stress, staining for the P-body marker GE1 showed increased numbers of prominent P-bodies in both cell lines as well as SGs containing GFPMBNL1, CUGBP1 and the canonical SG protein TIA1 (Figs 4B,C and 7D,E). In NaAsO_2_-treated cells of either cell line most P-bodies contained GFP-MBNL1 but GFPMBNL1 levels in P-bodies as a percentage of total cellular GFPMBNL1 was smaller in HeLa_CTG_960__GFPMBNL1 cells compared with HeLa_CTG_12__GFPMBNL1 control cells (0.08±0.1% compared with 0.1±0.1%, respectively) (Fig. 4F). As can be seen in Figs 4B and 8A, MBNL1 and CUGBP1 were better markers for SGs in these cells than TIA1, the latter also showing some staining around the plasma membrane. This was not seen with MBNL1, CUGBP1 or polyadenylated [poly(A)] RNA. Using superresolution airyscan microscopy, localisation of both GFPMBNL1 and CUGBP1 was revealed to be non-uniform within each SG and largely co-incident (Fig. 4D). In both cell lines colocalisation of GFPMBNL1 and CUGBP1, measured using the Pearson correlation coefficient, was very high. However, a statistically significant increase was seen in the HeLa_CTG_960__GFPMBNL1 cell line compared to the HeLa_CTG_12__GFPMBNL1 control line (Fig. 4E) (0.90±0.05 compared to 0.87±0.07, respectively), suggesting a subtle change in SG architecture with respect to the distribution of MBNL1 and CUGBP1, associated with the presence of CUGexp RNA.

### SG formation and dispersal are altered by CUGexp RNA expression in our inducible HeLa cell model

**Fig. 5.**
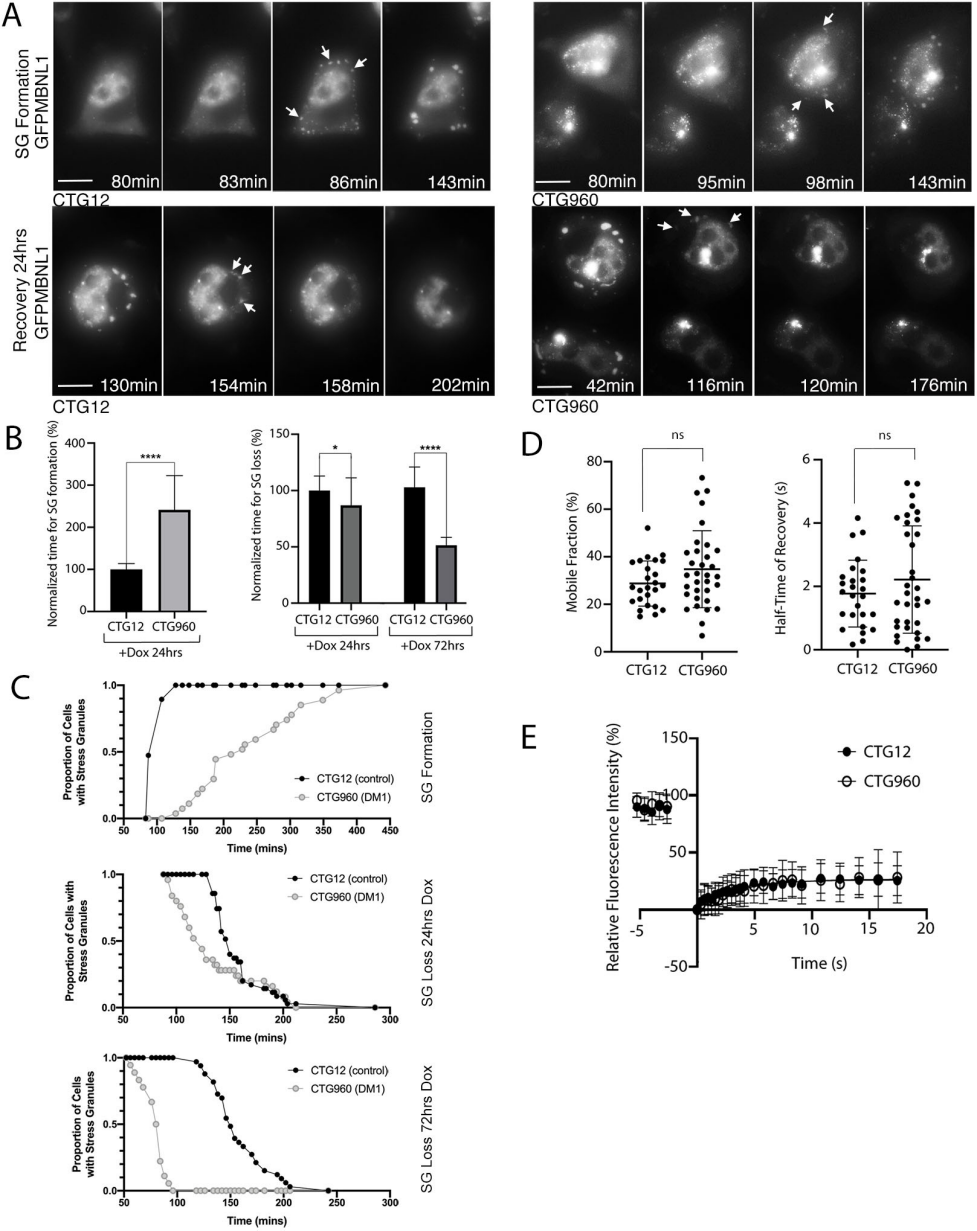
**Kinetics analysis of SGs reveals altered formation and dispersal of SGs in an inducible cell model of DM1, and increased variance in MBNL1 dynamics.** (A) Selected time-points from representative time-lapse images showing the formation (top) and dispersal during recovery (bottom) of SGs (arrows) in doxocycline (Dox)-induced CTG12 (left) and CTG960 (right) HeLa cell lines treated with NaAsO_2_ to induce SGs. The ‘formation time’ of SGs was recorded as the time-point at which at least five SGs were first detected. The ‘dispersal time’ of SGs was recorded as the first time-point at which SGs could no longer be clearly detected. Scale bars: 10 µm. See also Movies 1-4. (B) CTG12 control and CTG960 DM1 model cells were induced with Dox (+DOX), and time of SG formation (left) and SG loss (right) were analysed after treatment with NaAsO_2_ (analysed immediately for SG formation, and after 90 minutes of pre-treatment and drug removal for SG dispersal). Bar graphs show the time of SG formation after induction for 24 h (left) and time of SG loss after induction for 24 h or 72 h (right) in CTG960 cells normalised to the mean values measured in control (CTG12) cells (set as 100%). CTG12, *n*=19 cells; CTG960, *n*=27 cells for SG formation; ****P<0.0001. CTG12, *n*=35 cells; CTG960, *n*=25 cells for SG loss at 24 h; *P<0.05. CTG12 *n*=33; CTG960 *n*=18 cells for SG loss at 72 h; ****P<0.0001 unpaired *t*-tests. Each graph is representative of two independent experiments. (C) The proportion of cells containing SGs was plotted against time for the full time-course of treatment with or recovery from treatment with NaAsO_2_. This emphasises the difference in dynamics of SG formation (top) between cells expressing CTGexp RNA (DM1) and control RNA, but also the differences regarding SG loss when cells were Dox-induced for 24 h (centre) or 72 h (bottom). The increased difference seen with RNA induction for 24 h or 72 h confirms that these changes are due to CUGexp expression, rather than random variation between the cell lines. (D) The mobile fraction (left) and half-time of fluorescence recovery (right) of GFP-tagged MBNL1 (GFPMBNL1) in SGs is similar in CTG960 and CTG12 HeLa cells (Mann–Whitney *U* test; ns, not significant). (E) Plotted is the relative fluorescence recovery of GFPMBNL1 in SGs over time in response to photobleaching (488 nm laser, 2.5 s pulse), see also Fig. S7. Closed circles, SGs in HeLa_CTG_12__GFPMBNL1 cells (*n*=41); open circles, SGs in HeLa_CTG_960__GFPMBNL1 cells (*n*=25). All data are displayed as the mean±s.d.

To investigate further the effects the presence of CUGexp RNA has on GFPMBNL1-containing SGs, we used time-lapse imaging of CTG_960_- and CTG_12_-expressing cells during the response to and the recovery from stress induction following treatment with NaAsO_2_. First, HeLa_CTG_960__GFPMBNL1 and HeLa_CTG_12__GFPMBNL1 cells were induced for 24 h with Dox. Following addition of NaAsO_2_ (0.25 mM), time-lapse microscopy was carried out immediately and *z*-stacks of images were taken every 4 min until the formation of SGs was clearly observed (Fig. 5A). This revealed a pronounced delay in the formation of SGs in cells expressing CUGexp RNA compared with those that did not (HeLa_CTG_960__ GFPMBNL1, 36±12 min compared with HeLa_CTG_12__GFPMBNL1, 15±2 min, respectively) (Fig. 5B,C). This change was not seen when using endogenous MBNL1 and TIA1 to detect SGs in cells not induced with Dox (Fig. S7). As cellular stress induced by NaAsO_2_ is reversible and dispersal of SGs at least as important for their regulation as is their formation, we next investigated the dispersal time of GFPMBNL1-containing SGs in HeLa_CTG_960_GFPMBNL1 compared to control cells. Following induction with Dox for 24 h or 72 h cells were treated for 45 min with 0.5 mM NaAsO_2_ to ensure full induction of SGs in both lines. Upon thorough removal of NaAsO_2_, cells were imaged during recovery, with cells marked as recovered when no SGs were observed anymore (Fig. 5A). In contrast to the delay in formation of SGs associated with CUGexp RNA, GFPMBNL1-containing SG dispersal occurred more quickly in cells containing CUGexp RNA compared to control cells (HeLa_CTG_960__ GFPMBNL1, 133±38 min at 24 h and 79±11 min at 72 h; HeLa_CTG_12__GFPMBNL1, 158±30 min at 24 h and 159±28 min at 72 h). The increased speed of dispersal was more pronounced after 72 h than after 24 h (Fig. 5B,C). The disassembly seen when using MBNL1 as a marker for SGs closely resembled that reported previously when using GFP-G3BP1 as a marker in human U-2 OS osteosarcoma cells (Wheeler et al., 2016), with breakdown of large SGs into much smaller fragments (Movies 3 and 4). This suggested that we were visualising the disassembly of SGs, rather than simply the loss of MBNL1 from SGs, as the latter would be visualised as a gradually weakening signal in an SG of unchanged size. Taken together, these data show that the presence of the CUGexp RNA affects both SG formation in response to NaAsO_2_ treatment and SG dispersal upon stress removal, suggesting an overall defect in SG regulation associated with the presence of the CUGexp RNA characteristic of DM1.

### MBNL1 dynamics within SGs show subtle alterations in HeLa cells expressing CUGexp RNA

To investigate the effect of CUGexp RNA on the dynamics of MBNL1 within SGs, FRAP analysis was used on HeLa_CTG_12__GFPMBNL1 and HeLa_CTG_960__ GFPMBNL1 cells that had been induced with Dox for 24 h prior to treatment with NaAsO_2_ for 45 min (Fig. 5C-E). This analysis revealed a smaller mobile fraction and shorter half-time of recovery for GFPMBNL1 in HeLa cells compared to HLECs (Fig. 2), suggesting GFPMBNL1 is less able to exchange within SGs in HeLa cells compared to HLECs (mobile fraction 28.7±9.5% for HeLa_CTG_12__GFPMBNL1 and 34.8±16.2% for HeLa_CTG_960__ GFPMBNL1; half-time of recovery 1.8±1.1 s for HeLa_CTG_12__GFPMBNL1 and 2.2±11.7 s for HeLa_CTG_960__ GFPMBNL1). No statistically significant differences in the mobile fraction of GFPMBNL1 or half-time of recovery between the two HeLa cell lines were detected (Fig. 5D). Visualisation of individual data sets, however, suggested increased variability in the data obtained for the HeLa_CTG_960__ GFPMBNL1 compared to the CTG12 control cell line, with cells containing CUGexp repeats showing an apparent split of their half-time of recovery measurements (Fig. 5D). This idea was supported by an increased variance in the half-time of recovery values for the CTG960 line (76.5%) compared with the CTG12 line (59.6%). Furthermore, a D'Agostino–Pearson omnibus normality test confirmed non-normal distribution of the half-time of recovery of GFPMBNL1 in SGs in HeLa_CTG_960__ GFPMBNL1 cells (*P*=0.0084), whereas in the control line the data were normally distributed.

### Reduction of MBNL1 or CUGBP1 expression alters the respective dynamics of CUGBP1 or MBNL1 in SGs

**Fig. 6.**
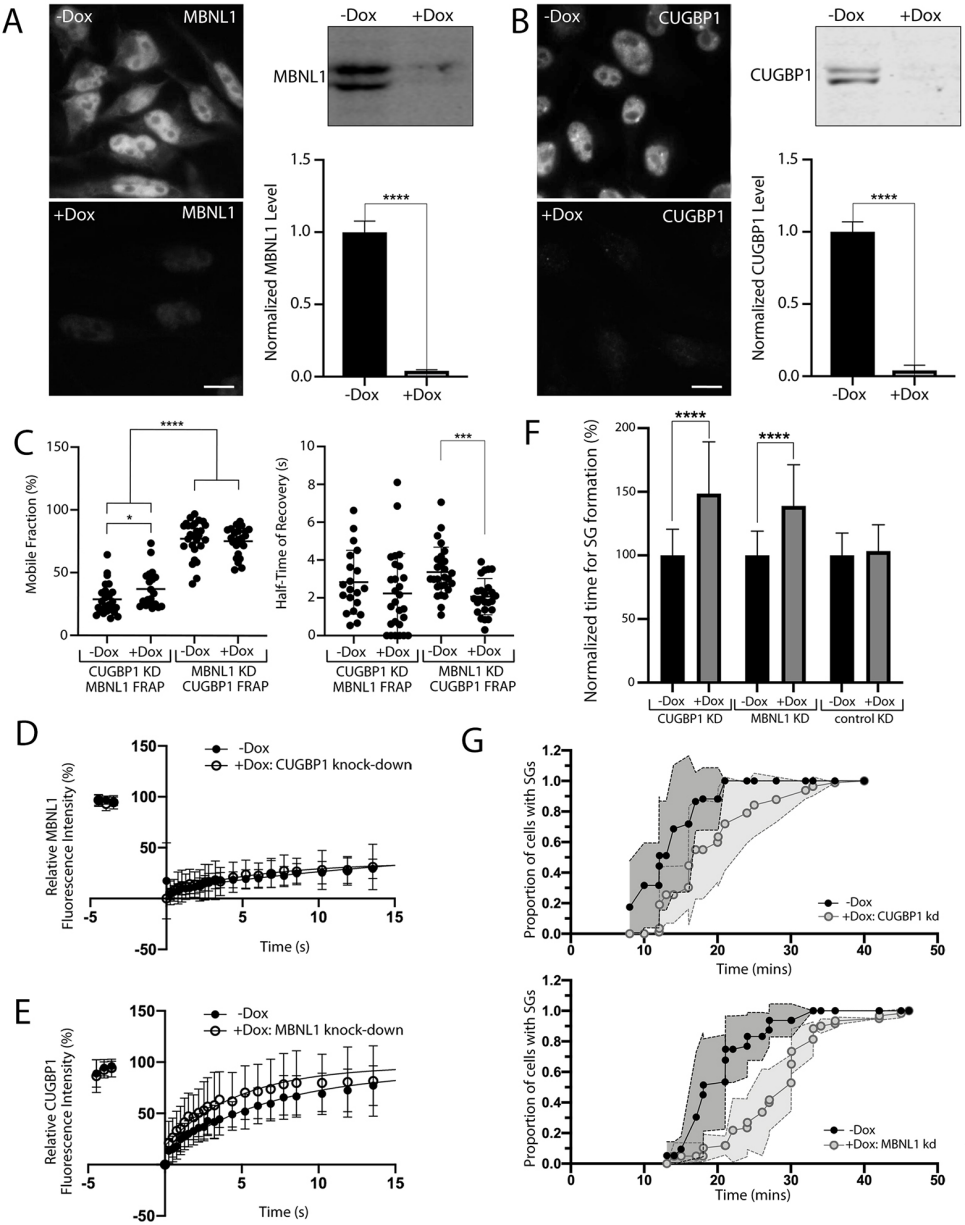
**SG dynamics are altered by reduction in expression of MBNL1 or CUGBP1.** (A,B) Stable cells containing Dox-inducible shRNAs targeting MBNL1 (A) or CUGBP1 (B) were treated with (+) or without (–) Dox. Levels of MBNL1 or CUGBP1 were validated using antibodies against endogenous MBNL1 and endogenous CUGBP1, respectively, in immunocytochemistry (left) and immunoblotting (right) experiments. Scale bars: 10 µm, ****P<0.0001 (unpaired *t*-test, data from three independent experiments each). (C) Mobile fractions (left) and half-time of fluorescence recovery (right) of GFP-tagged MBNL1 or GFP-tagged CUGBP1 in SGs of +Dox or –Dox cells with induced knockdown (KD) of either CUGBP1 or MBNL1. **** *P*<0.0001, *P<0.05, ***P<0.001 (Mann–Whitney *U* test). (D) Curve fit for FRAP of GFP-tagged MBNL1 in SGs of +Dox cells with CUGBP1 knockdown (closed circles, *n*=21 cells) compared to that of −Dox control cells (open circles, *n*=27 cells). (E) Curve fit for FRAP of GFP-tagged CUGBP1 in SGs of +Dox cells with MBNL1 knockdown (closed circles, *n*=24) compared to that of −Dox control cells (−Dox, open circles, *n*=27). (F) Time of SG formation in response to NaAsO_2_ in cell lines with Dox-inducible CUGBP1 and MBNL1 knockdown compared to a negative control, normalised to the mean values for each cell line without knockdown (-Dox), set at 100%. ****P<0.0001 (unpaired *t*-tests. For CUGBP1 KD -DOX: *n*=96; +DOX: *n*=107; for MBNL1 KD -DOX: *n*=56; +DOX: *n*=60; for control (luciferase) KD -DOX: *n*=20; +DOX: *n*=14). Data shown are pooled from three independent experiments. (G) Plotted is the proportion of SG-containing cells over time for the full time-course of SG formation, emphasising the difference in the dynamics of SG formation in cells with reduced CUGBP1(top) or MBNL1(bottom) levels compared to that of control cells. Grey shading indicates ±s.d. for the curves it surrounds. All data are displayed as the mean±s.d.

Having established that MBNL1 and CUGBP1 colocalise closely in SGs, and that there may be subtle changes in the dynamics of MBNL1 in SGs associated with the presence of CUGexp RNA, we next sought to investigate the relationship between these two DM1-associated proteins in SGs. Stable cell populations were established containing Dox-inducible lentiviral shRNAs targeting MBNL1 (HeLa_pLKO_MBNL1) or CUGBP1 (HeLa_pLKO_CUGBP1). Robust reduction of MBNL1 (Fig. 6A) or CUGBP1 levels (Fig. 6B) following shRNA induction by Dox, were validated using both immunocytochemistry and immunoblotting. FRAP analysis of transiently expressed GFP-tagged CUGBP1 or GFPMBNL1 in, respectively, HeLa_pLKO_MBNL1 or HeLa_pLKO_CUGBP1 cells was then used to compare the dynamics of the two proteins in SGs and to assess the effects of the reciprocal knockdown.

In unmanipulated cells, CUGBP1 had a significantly larger mobile fraction than MBNL1 (77.2±14.5% for CUGBP1 versus 28.8±12.3% for MBNL1) but a comparable half-time of recovery (compare data sets; −Dox in Fig. 6C). Reduced MBNL1 expression did not alter the mobile fraction of GFP-tagged CUGBP1 but resulted in decreased half-time of recovery, indicating that a similar amount of CUGBP1 was free to exchange within the SGs but that its rate of movement was faster (Fig. 6C,E). In contrast, reduction in CUGBP1 expression resulted in a slight increase in the mobile fraction for GFPMBNL1, without altering the half-time of recovery, indicating that a larger amount of GFPMBNL1 was free to exchange within the SGs but with the same rate of movement (Fig. 6C,D). These data suggest a complex relationship between MBNL1 and CUGBP1 in SGs, with either protein having some effect on the dynamics of the other.

Time-lapse microscopy was then used to assess the kinetics of recruitment of MBNL1 or CUGBP1 into SGs in response to treatment with NaAsO_2_ in HeLa cells expressing reduced levels of CUGBP1 or MBNL1, respectively (Fig. 6F,G). For both proteins, reduction in expression resulted in a delay in recruitment of the reciprocal protein into SGs. This delay was not seen in control cells expressing luciferase-targeting shRNAs. For MBNL1 knockdown, the delay in SG formation was also markedly less (38.9±32.4%) compared with that seen following CUGexp induction (141.6±81.1) (Fig. 5B), despite the high efficiency of MBNL1 reduction, suggesting that lack of MBNL1 is not the sole cause of the alterations to rates of SG formation observed in the DM1-inducible HeLa model.

### P-bodies and SGs both appear as LLPS structures in HLECs

**Fig. 7.**
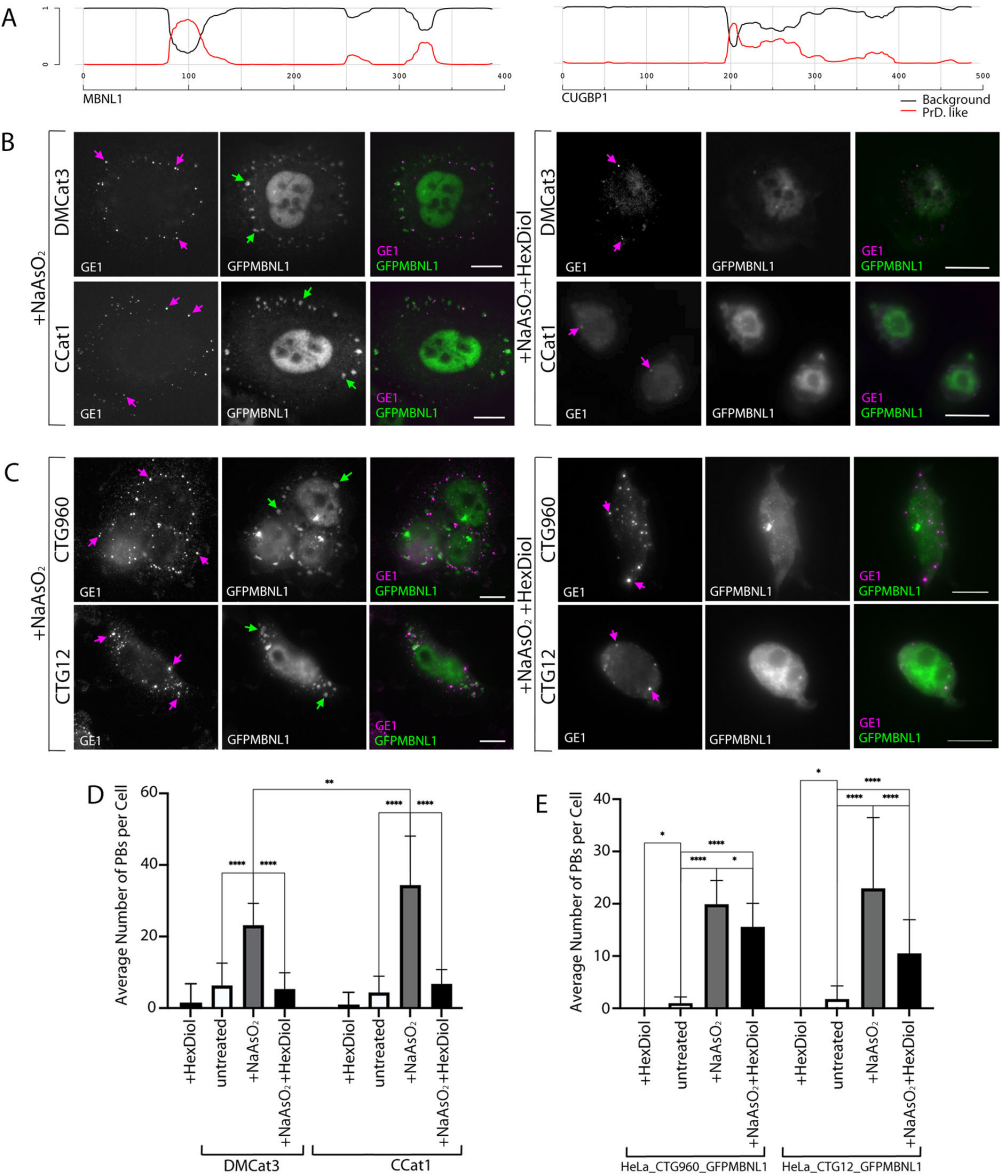
**P-bodies and SGs both show characteristics of LLPS structures, with PB stability increased by induction of CTGexp repeats.** (A) Graph showing the predicted PrLDs for MBNL1 (left) and CUGBP1 (right), generated using PLAAC software (Lancaster et al., 2014). Parameters used were a core length of 30 amino acids and a background frequency for homo sapiens (see Materials and Methods). PrLDs are depicted where the probability is >0.5 on the *y*-axis. Red lines, PrLD probability score; black line, background probability score. (B) DM1 patient (DMCat3, top row) and control (CCat1, bottom row) HLECs (see: ‘Cell lines and cell culture’ under Materials and Methods) treated with NaAsO_2_ (left) or with NaAsO_2_ followed by 1,6-hexanediol (right). SGs (green arrows) containing GFP-tagged MBNL1 (GFPMBNL1; green in merged images) are clearly seen cells treated NaAsO_2_ alone but absent in cells treated with NaAsO_2_ followed by 1,6-hexanediol. P-bodies (magenta arrows) detected with antibodies against endogenous GE1 (magenta in merged images) are numerous in cells treated with NaAsO_2_ alone but less numerous in cells treated with NaAsO_2_ followed by 1,6-hexanediol. Scale bars: 10 µm. (C) HeLa_CTG_960__GFPMBNL1 cells treated with NaAsO_2_ (left) or with NaAsO_2_ followed by 1,6-hexanediol (right). SGs (green arrows) containing GFPMBNL1 (green in merged images) are clearly seen in NaAsO_2_-treated cells but absent in cells treated with NaAsO_2_ followed by 1,6-hexanediol. P-bodies (magenta arrows) detected with antibodies against endogenous GE1 (magenta in merged images) are numerous in cells treated with NaAsO_2_ alone but less numerous in cells treated with NaAsO_2_ followed by 1,6-hexanediol. Scale bars: 10 µm. (D) Shown are the average number of P-bodies per cell in DM1 (DMCat3) and control (CCat1) HLECs treated with 0.5 mM NaAsO_2_ or treated with 0.5 mM NaAsO_2_ followed by 5% 1,6-hexanediol compared to untreated cells. *n*=53 (DMCat3), *n*=53 (CCat1); **P<0.05, ****P<0.0001; one-way ANOVA, pooled from three independent experiments in each case. All data are displayed as the mean±s.d. (E) P-bodies per cell in HeLa cells with (HeLa_CTG_960__GFPMBNL1) and without (HeLa_CTG_12__GFPMBNL1) CTGexp foci treated with 0.5 mM NaAsO_2_ or treated with 0.5 mM NaAsO_2_ followed by 5% 1,6-hexanediol compared to untreated cells. *P<0.05, ****P<0.0001. *n*=108 (HeLa_CTG_960__GFPMBNL1); *n*=69 (HeLa_CTG_12__GFPMBNL1); Kruskal–Wallis, pooled from three independent experiments in each case. All data are displayed as the mean+s.d.

In mammalian cells, both SGs and P-bodies are thought to be LLPS structures, formed as a result of weak promiscuous interactions between low-complexity prion-like domains (PrLDs) found in their key protein components (Jain et al., 2016; Kroschwald et al., 2015; Protter and Parker, 2016). The rapid exchange of MBNL1 and CUGBP1 detected by FRAP (Figs 2, 5 and 6) within SGs and P-bodies, and the ability of SGs to fuse and separate (Fig. 5 and Movies 1-4) as seen in our two models of DM1, are consistent with this hypothesis. To determine whether MBNL1 and CUGBP1 contain PrLDs that are likely to contribute to the formation of LLPS structures, we analysed their amino acid (aa) sequences by using the prion-like amino acid composition (PLAAC) software (http://plaac.wi.mit.edu/; Lancaster et al., 2014) designed to predict PrLDs. This analysis revealed probable PrLDs in both proteins, with that in MBNL1 being longer than that in CUGBP1 (Fig. 7A).

Having identified subtle changes in the dynamic interaction of MBNL1 with SGs and P-bodies associated with the presence of CUGexp RNA as well as a PrLD in MBNL1, we next sought to investigate the behaviour of SGs and P-bodies in response to the solvent 1,6-hexanediol, previously shown to disrupt LLPS structures in yeast and mammalian cells (Kroschwald et al., 2015). To determine whether SGs and P-bodies behave as LLPS structures in HLECs, cells were pre-treated with NaAsO_2_, followed by treatment with 5% 1,6-hexanediol for 20 min. All SGs, detected with antibodies against endogenous MBNL1, dispersed upon 1,6-hexanediol treatment of DM1 and control HLECs (Fig. 7B). This indicates that SGs in these cells show the liquid-like property of dispersal following treatment with1,6-hexanediol, in addition to the rapid exchange of their protein components and their ability to fuse and split. This is consistent with analysis of the biophysical properties of SGs in other mammalian cell lines. The number of P-bodies, detected with antibodies against endogenous GE1, was comparable in DM1 and control HLECs, and treatment of unstressed cells with 1,6-hexanediol caused an almost complete loss (Fig. 7D). Initial treatment with NaAsO_2_ significantly increased P-body numbers in both DM1 and control HLECs, with their numbers significantly lower in DM1 compared to control cells (Fig. 7B,D). Subsequent treatment with 1,6-hexanediol resulted in a marked reduction in the number of P-bodies per cell back to a level comparable to that seen in unstressed cells in both DM1 and control cells (Fig. 7B,D). This suggests that the majority of NaAsO_2_ stress-induced P-bodies in HLECs are formed by LLPS, in common with those seen in unstressed cells; however, a minority of P-bodies might represent more-stable structures showing insensitivity to 1,6-hexanediol treatment.

### NaAsO_2_ stress-induced P-bodies in HeLa cells that express CTGexp are resistant to treatment with 1,6-hexanediol

Further investigation of the characteristics of P-bodies in stressed cells was made using the inducible HeLa cell model, allowing a more direct assessment of any effect the presence of CUGexp RNA has in a genetically homogeneous background. Exposure of cells from lines HeLa_CTG_12__GFPMBNL1 and HeLa_CTG_960__GFPMBNL1 to 1,6-hexanediol without pre-treatment with NaAsO_2_ resulted in complete P-body dispersal in both lines, consistent with the liquid-like properties for P-bodies in unstressed cells (Fig. 7E). Treatment with NaAsO_2_ resulted in an increased number of P-bodies, similar to that seen in HLECs (Fig. 7C,E). Subsequent treatment with 1,6-hexanediol significantly reduced P-body numbers in both cell lines but, in contrast to the results from HLECs, the number of P-bodies per cell did not return to pre-stressed levels (Fig. 7C,E). Notably, the average number of P-bodies per cell remained higher in cells containing CUGexp RNA than in those without. Taken together, these data suggest that the P-bodies present in NaAsO_2_-stressed cells represent a diverse population with differing degrees of sensitivity to 1,6-hexanediol. Furthermore, the increase in 1,6-hexanediol-resistant P-bodies in HeLa_CTG_960__GFPMBNL1 suggests that the presence of CUGexp RNA pre-disposes cells to the formation of more-stable P-bodies when subjected to stress.

### SGs in HeLa cells expressing CTGexp contain less poly(A) RNA and show altered docking events with P-bodies, suggesting defects in SG function

**Fig. 8.**
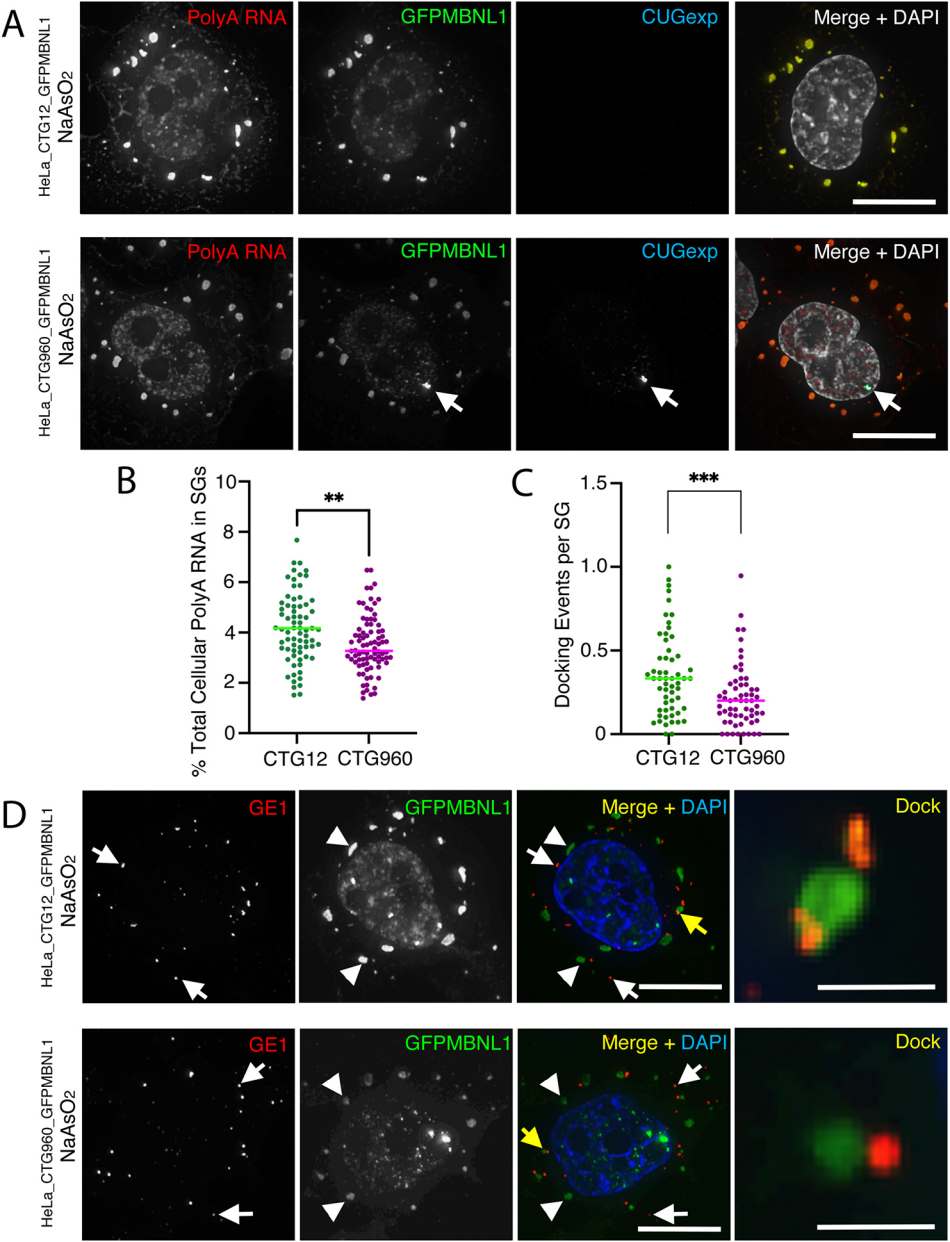
**Reduced amounts of poly(A) RNA in SGs, and altered docking events between P-bodies and SGs suggests defects in their function in cells expressing CUGexp RNA** (A) RNA FISH of HeLa_CTG12_GFPMBNL1 (top) and HeLa_CTG960_GFPMBNL1 (bottom) cells treated for 90 min with NaAsO_2_. Poly(A) RNA (first images; red in merged images) accumulates in the nucleus and in cytoplasmic SGs of both cell lines. CUGexp RNA (third images; blue in merged images) is detected only in the nuclear foci (arrows) of HeLa_CTG960_GFPMBNL1 cells. GFP-tagged MBNL1 (GFPMBNL1) accumulates in SGs of both cell lines and in the CUGexp foci when they are present (second images; green in merged images). (B) The total amount of cellular poly(A) RNA (in %) in SGs is reduced in cells that express CUGexp RNA (CTG960) than in control cells (CTG12). Data shown are pooled from two independent experiments. *n*=71 (CTG12), *n*=84 (CTG960); **P<0.01, unpaired *t*-test. (C) The number of docking events between SGs and P-bodies is lower in cells that express CUGexp RNA (CTG960) than in control cells (CTG12). Data shown are pooled from two independent experiments (CTG12, *n*=57 cells; CTG960, *n*=60 cells); ***P<0.001, Mann–Whitney *U* test. (D) HeLa_CTG12_GFPMBNL1 (top) and HeLa_CTG960_GFPMBNL1 (bottom) cells, treated with NaAsO_2_ for 90 min. P-bodies detected with antibodies against GE1 (indicated by white arrows in first and third images; shown in red in merged and magnified images), show docking events (indicated by yellow arrows in merged images and shown magnified in panels on the right). SGs are detected by staining for GFPMBNL1 (indicated by arrowheads and shown in green in merged images). All nuclei are counterstained with DAPI (white in merged image in A; blue in merged image in B). Scale bars: 10 µm (A,D); 2 µm (magnified images in D).

The main function of SGs is the temporary sequestration of untranslated mRNA; so, we next sought to address the consequences of CUGexp repeat RNA for the recruitment of poly(A) RNA into SGs. Dox-induced cells from lines HeLa_CTG_12__GFPMBNL1 and HeLa_CTG_960__GFPMBNL1 were treated with NaAsO_2_, and FISH to detect poly(A) RNA was carried out using a PolydT probe alongside a probe for CUGexp RNA (Fig. 8A). The percentage of total cellular poly(A) RNA in SGs (identified by using GFPMBNL1) was decreased in cells expressing CUGexp RNA compared to control cells (Fig. 8B) (3.5±1.1% for CTG960 versus 4.2±1.4% for CTG12). This occurred despite the absence of CUGexp RNA from SGs themselves. Close physical interactions or docking events between SGs and P-bodies are often seen. The reason for these is unclear, but it has been proposed that, through these events, mRNPs can be exchanged between SGs and P-bodies (Protter and Parker, 2016). Detection of P-bodies by using antibody against endogenous GE1 in NaAsO_2_-treated cells (Fig. 8D) revealed decreased incidences of such docking events per SG associated with the presence of CUGexp RNA (Fig. 8C) (0.21±0.17 for CTG960 versus 0.36±0.25 for CTG12). The lower amounts of poly(A) RNA within SGs in cells that express CUGexp RNA suggest a functional defect in SGs, which may be partially explained by reduced number of docking events between P-bodies and SGs.

## DISCUSSION

### DM1-associated proteins MBNL1 and CUGBP1 are both components of P-bodies and SGs

In DM1 the activities of two multi-functional RNA-binding proteins, MBNL1 and CUGBP1, are altered. MBNL1 is recruited to aberrant nuclear CUGexp foci that are the hallmark of DM1, while the activity of CUGBP1 is upregulated. There is evidence that this leads to changed alternative splicing patterns in DM1, with MBNL1 and CUGBP1 acting antagonistically as splicing regulators (de Haro et al., 2006; Konieczny et al., 2014; Pettersson et al., 2015; Wang et al., 2015). The full extent of the effects of DM1 on RNA production and turnover within the cell, however, are not understood.

Following our observation that MBNL1 and CUGBP1 colocalise in small punctate cytoplasmic structures, despite showing no obvious colocalisation within the nucleus, we investigated the localisation and dynamic behaviour of MBNL1 and CUGBP1 in the cytoplasm in two independent cell culture models of DM1. In HLECs from DM1 patients and controls (Fig. 1) MBNL1 and CUGBP1 colocalise in both P-bodies in cells grown under normal conditions and in and SGs following NaAsO_2_ treatment. P-bodies and SGs are closely related structures involved in cytoplasmic mRNA regulation, and there is some evidence that components of P-bodies relocate to SGs in stressed cells (Anderson et al., 2015; Protter and Parker, 2016; Shah et al., 2016). In our novel inducible HeLa cell model of DM1 (Figs 3 and 4), the situation is slightly more complex. CUGBP1 and MBNL1 colocalise in SGs following NaAsO_2_ treatment but, in unstressed cells – in which CUGBP1 accumulates within P-bodies regardless of the presence of CUGexp RNA – MBNL1 is largely absent from P-bodies of cells that contain CUGexp foci within their nuclei. This observation suggests that the role of MBNL1 in P-bodies is disrupted by the presence of CUGexp RNA.

### SGs are not detected in HLECs from DM1 patients or in an inducible DM1 HeLa model cell line grown under normal conditions

In contrast to previous reports of SGs in DM1 myoblasts under normal growth conditions (Huichalaf et al., 2010; Ravel-Chapuis et al., 2016), suggesting that DM1 leads to a higher basal level of stress, we did not see evidence of SGs in either of our cell models grown under standard culture conditions. The reason for this is unclear but the difference is unlikely to be a simple correlation with the size of the CUGexp foci that, although small in HLECs from DM1 patients (Coleman et al., 2014), are large in our inducible HeLa cell model. The difference might reflect the use of different cell types and highlights the complexity of the cellular pathology of DM1, a multi-systemic condition with highly variable symptoms. DM1 patients are highly vulnerable to cataract development. Lens epithelial cells act as stem cells for lens development throughout life (Smith and Gutmann, 2016); so, increased basal stress in HLECs could be involved in DM1-associated cataract formation. Our results, however, suggest that a basal stress level sufficient to induce SGs is not present in DM1 HLEC cell lines. It is possible, though, that an underlying increased basal stress response is present in cells of the lens at later stages of differentiation, *in vivo* only or at levels insufficient to induce SGs.

### SG turnover is altered in cell culture models of DM1

DM1 HLECs treated with NaAsO_2_ to induce oxidative stress showed no increase in the rate of SG formation compared to age-matched controls (Fig. 2). In our novel HeLa cell model that contains larger CUGexp foci than DM1 patient-derived HLECs, a pronounced delay in SG formation was seen (Fig. 5). Impaired SG formation, in the absence of a detectable underlying stress response, has been reported previously in fibroblasts from DM1 patients, induced to adopt a myoblast phenotype by exogenous expression of MyoD (Ravel-Chapuis et al., 2016), but not in the unmanipulated fibroblasts. This was interpreted as a cell type difference as myoblasts – but not fibroblasts – are affected in DM1. However, nuclear foci of the myoblast cells also were larger compared to those of the parental fibroblasts. Our new results are in broad agreement with these observations, and further suggest a complex interplay between cell type and size of CUGexp foci affecting cellular responses to stress.

In contrast to the delay seen in SGs formation, both of our cell models showed increased rates of SG dispersal in cells containing the CUGexp RNA when stress was removed. In the inducible HeLa cell model, this effect was more pronounced when CUGexp foci were induced for longer periods of time (72 h versus 24 h) (Fig. 5B), suggesting that the changes are directly linked to the duration of expression of CUGexp RNA. SGs are transient structures, with the mRNAs sequestered within them destined to return to active translation. Dispersal of SGs is a tightly regulated process, sometimes involving a form of autophagy (Hardy et al., 2017; Mateju et al., 2017; Protter and Parker, 2016; Turakhiya et al., 2018). The rapid loss of SGs from cells containing CUGexp foci may indicate alterations or impairment in the mechanisms required for regulated SG dispersal associated with the presence of CUGexp RNA. This would require considerable additional analyses but may be of importance for understanding cataract development associated with DM1. Defects in autophagy are associated with cataract development, although the mechanisms and types of autophagy involved are, as yet, unclear (Costello et al., 2013; McWilliams et al., 2019; Morishita et al., 2013; Morishita and Mizushima, 2016).

### Alterations to SG turnover cannot solely be attributed to the sequestration of MBNL1 in CUGexp foci

The sequestration of MBNL1 in CUGexp foci has long been cited as a main cause of cellular defects in DM1. However, we have previously reported that the amount of total cellular MBNL1 present within nuclear foci in HLECs from DM1 patients is extremely small, i.e. <0.2% (Coleman et al., 2014). Even in our HeLa cell model, which appears to contain large and very bright nuclear foci when viewed using GFPMBNL1, the amount of cellular GFPMBNL1 contained in the foci is <1% (Fig. 3C). Delayed formation of SGs was also seen in HeLa cells without CUGexp foci but with shRNA-mediated MBNL1 depletion (Fig. 6). While this does implicate loss of MBNL1 as a direct cause of disruption to SG formation, the delay seen following MBNL1 reduction by shRNA was much less pronounced than that resulting from CUGexp foci induction, despite the almost complete loss of MBNL1 seen in shRNA-treated cells. The small effect on SG turnover of almost complete MBNL1 depletion compared to that seen in cells containing CUGexp foci, in which only very small amounts of MBNL1 are mislocalised to foci, strongly suggests that sequestration of MBNL1 in CUGexp foci is not the primary cause of the alterations to SG turnover. It is likely, though, that MNBL1 is also bound to CUG repeats in *DMPK* mRNA outside nuclear foci.

### MBNL1 and CUGBP1 are likely to be core components of SGs and might be recruited from P-bodies

MBNL1 and CUGBP1 have different dynamics within SGs, with GFP-tagged CUGBP1 showing both a larger mobile fraction and faster recovery in FRAP experiments than GFPMBNL1 (Fig. 6). Previous studies of various SG proteins in different cell types have reported highly divergent dynamic behaviours (Bley et al., 2015), with proteins essential for SG formation exhibiting faster kinetics within SGs – i.e. with half-time of recovery between 2 s and 3 s – and larger mobile fractions than those dispensable for their formation. MBNL1 exhibits behaviour similar to that published for TIA1, a well-established SG marker whose depletion results in impaired SG formation (Bley et al., 2015; Gilks et al., 2004). Together with the delayed formation of SGs in cells with disrupted MBNL1 expression, this suggests that MBNL1 is important for SG integrity. CUGBP1 – although exhibiting a rapid exchange within SGs similar to that of MBNL1 – has a small mobile fraction previously reported to be characteristic of non-essential SG components (Bley et al., 2015). Reduction of CUGBP1 expression, however, also caused a delay in SG formation, which would not be expected for a non-essential component, emphasising further the complexity of these structures.

It has been suggested that SGs in mammalian cells are composed of a mixture of liquid and solid phases (Protter and Parker, 2016) with a solid core structure surrounded by a more dynamic shell (Jain et al., 2016; Markmiller et al., 2018; Protter and Parker, 2016). High-resolution airyscan microscopy reveals close colocalisation of MBNL1 and CUGBP1 in SGs, with both proteins showing punctate accumulations similar to those described as G3BP-containing SG core by Jain et al. (2016) using STORM microscopy. Moreover, CUGBP1 has previously been identified in the proteome of SG core structures (Jain et al., 2016; Kuechler et al., 2020; Youn et al., 2018), which strengthens the evidence that the two DM1-associated proteins MBNL1 and CUGBP1 are core SG proteins. This represents a slightly unusual situation, as proteins required for the formation of a particular RNP structure are usually confined to that structure (reviewed by Corbet and Parker, 2019), whereas MBNL1 and CUGBP1 are also found in P-bodies as well as in specific nuclear regions (Coleman et al., 2014; Fujimura et al., 2008).

In our inducible HeLa cell model of DM1, the presence of CUGexp RNA resulted in a slight decrease in the Pearson correlation coefficient regarding the colocalisation of GFPMBNL1 and endogenous CUGBP1 within SGs, suggesting an alteration in the interactions of these two proteins with SGs. In the same unstressed cells, both CUGBP1 and GFPMBNL1 were detected within P-bodies when CUGexp repeats were not expressed; however, GFPMBNL1 was absent from P-bodies in cells that did express CUGexp repeats. Upon NaAsO_2_-induced stress, GFPMBNL1 was seen in a subset of P-bodies in both our DM1 cell lines, but the amount of GFPMBNL1 in P-bodies was significantly reduced in cells with CUGexp foci. It is conceivable that this loss of MBNL1from P-bodies – as a core SG component – contributes to the delayed formation of SGs as seen in these cells following treatment with NaAsO_2_.

### Alterations to LLPS might be associated with DM1

PrLDs within low-complexity sequences promote the formation of LLPS structures, including mammalian SGs (Ditlev et al., 2018; Markmiller et al., 2018; Protter and Parker, 2016). Analysis of the sequences of MBNL1 and CUGBP1 (Fig. 7) reveals small predicted PrLDs in both MBNL1 and CUGBP1, which might account for their propensity to accumulate SGs. The domain in MBNL1 is longer (28 aa) than that in CUGBP1 (10 aa).

Induction of CUGexp RNA in our DM1 HeLa cell model changed the distribution of the GFPMBNL1 half-time of recovery values, showing a subset of SGs with a much slower rate of recovery. The explanation for this is unclear but this reduced rate might be due to increased tendency for the formation of more solid SGs in cells containing nuclear foci. However, in cells with MBNL1 reduced to very low levels by using shRNA (Fig. 6), we observed an increase in the rate of CUGBP1 exchange within SGs, suggesting an increased fluidity of the structures overall. This apparent contradiction, taken together with the differences in rates of exchange of MBNL1 and CUGBP1 in SGs despite their close colocalisation, suggest that the dynamics of LLPS structures are extremely complex (reviewed by A and Weber, 2019) and that FRAP of individual constituent proteins is not a perfect method to examine the biophysical nature of cellular structures. It also provides further evidence that alterations to SGs associated with DM1 are not entirely due to loss of MBNL1.

1,6-Hexanediol has been extensively used to disrupt structures predicted to form by LLPS, *in vitro* and *in vivo* (Kroschwald et al., 2015; Molliex et al., 2015; Patel et al., 2007; Ribbeck and Gorlich, 2002; Updike et al., 2011). As with FRAP, this is not a fail-safe way to assess the LLPS nature of cellular structures, as the solvent is toxic to mammalian cells at higher concentrations (Wheeler et al., 2016). Treatment of HLECs and inducible HeLa cell models of DM1 with 1,6-hexanediol resulted in complete loss of SGs, as would be expected for a phase-separated structure. The effect on P-bodies was more complex. In unstressed cells, virtually no P-bodies were visible after treatment with 1,6-hexanediol. However, in HeLa cells pre-treated with NaAsO_2_ large numbers of additional P-bodies were formed during the stress response and a proportion of these remained following 1,6-hexanediol treatment, suggesting that they had lost their liquid-like properties. Of particular interest for understanding DM1 is that the number of 1,6-hexanediol-resistant P-bodies was significantly higher in HeLa cells containing CUGexp nuclear foci. This suggests that the presence of the DM1-associated CUGexp RNA influences the biophysical characteristics of P-bodies in stressed cells, perhaps as a result of the loss of MBNL1 from them.

### Functional defects might occur in SGs in cells containing CUGexp RNA

P-bodies and SGs show significant overlap in their components (reviewed by Youn et al., 2019), and it has been known for some time that SGs and P-bodies are closely linked structures that can dock to and fuse with each other, potentially facilitating the exchange of mRNPs between them (reviewed by Stoecklin and Kedersha, 2013). We now show that MBNL1 is a component of both SGs and P-bodies, and is partially lost from P-bodies in DM1 model HeLa cells that express CUGexp RNA (Fig. 4 and Fig. S6). Docking events between SGs and P-bodies are also reduced in these cells compared to controls, as is the proportion of total cellular poly(A) RNA present in SGs. This suggests that the function as well as dynamics of SGs is compromised in the presence of the CUGexp RNA that causes DM1. This occurs despite the absence of CUGexp RNA from SGs (Fig. 8) and might be mediated through altered biophysical interactions between P-bodies and SGs.

### Implications of altered SG and P-body dynamics for DM1 pathology

Defects in the normal physiology of cytoplasmic LLPS structures, particularly SGs, have been widely implicated in human diseases. The spontaneous conversion of PrLDs into an aggregated state, and subsequent transition from liquid to solid to form solid RNP aggregates has been proposed as a pathological mechanism in conditions including ALS and FTD (Li et al., 2013; Ramaswami et al., 2013). The presence of aberrant or persistent SGs is also associated with the cellular pathology of age-related diseases, neurodegeneration and some cancers (Mateju et al., 2017; Protter and Parker, 2016; Turakhiya et al., 2018). Furthermore, increased basal stress levels and defects in cellular stress responses have been suggested to occur in DM1 (Huichalaf et al., 2010; Ihara et al., 1995; Kumar et al., 2014; Toscano et al., 2005; Usuki and Ishiura, 1998; Usuki et al., 2000). Here, we report delayed SG formation and premature SG loss in cell culture models of DM1, together with altered biophysical characteristics of the closely related P-bodies. This strongly suggests that the presence of CUGexp RNA, perhaps partly as a consequence of the sequestration of MBNL1 by the CUG expansion within nuclear foci or elsewhere, results in defects in cellular responses to stress. The assembly and disassembly of SGs are complex, regulated processes (Wheeler et al., 2016). Failure to disassemble SGs in a controlled manner could impair the ability of cells to respond effectively to repeated stress events and, furthermore, might suggest a defect in autophagy associated with DM1. Defects in autophagy have recently been implicated in several degenerative conditions (Ranganathan et al., 2020) and autophagy is known to contribute to the generation of optically transparent lens fibre cells during differentiation of the lens epithelial stem cell population (Costello et al., 2013; Morishita et al., 2013; Morishita and Mizushima, 2016).

In summary, our data show alterations to the structure and regulation of SGs and P-bodies – two key LLPS structures involved in cytoplasmic mRNA metabolism and cellular stress responses in cell culture models of DM1. These changes are associated with the presence of CUGexp RNA and might be linked to deficiency of MBNL1 resulting from its accumulation in the nuclear CUGexp foci characteristic of DM1. However, with only small amounts of MBNL1 colocalising with nuclear CUGexp foci, and the failure of almost complete MBNL1 knockdown to recapitulate the magnitude of effects seen in cells expressing CUGexp RNA, MBNL1 sequestration in CUGexp foci is unlikely to provide a full explanation. Our observations point to a complex interplay between nuclear and cytoplasmic structures involved in mRNA metabolism, and suggests that further investigation is warranted regarding the contribution of both altered LLPS and autophagy towards cellular defects in DM1.

## MATERIAL AND METHODS

### Plasmids and lentiviral vectors

Plasmids pBI-Tet-CTG12 and pBI-Tet-CTG960 (Lee et al., 2012; Philips et al., 1998) were a gift from Prof. Thomas Cooper, Baylor College of Medicine, Houston. These constructs contain a bi-directional tetracycline-responsive promoter allowing the simultaneous expression of GFP and a *DMPK1* minigene containing an interrupted CTG repeat length corresponding to a healthy control state (pBI-Tet-CTG12) or a disease-relevant expansion (pBI-Tet-CTG960). To generate plasmids pBI-Tet-CTG12_GFPMBNL1 and pBI-Tet-CTG960_GFPMBNL1, sequence and ligation independent cloning (SLIC) was used to introduce the GFPMBNL1 cDNA sequence into the pBI-Tet-CTG12 vector, after which the disease-relevant expansion was introduced by restriction digest and ligation. To generate plasmid pcDNA3rtTA3, the cDNA sequence encoding the third generation rtTA protein (rtTA3) was obtained by PCR from plasmid MXS_PGK::rtTA3-bGHpA (Addgene plasmid #62446) (Sladitschek and Neveu, 2015) using primers 5′-gctagtaagcttatgtctagactggacaagagcaaagtc-3′ and 5′-atcatggatccttacccggggagcatgtc-3′ and cloned into the pcDNA3 vector for mammalian expression.

The cDNA sequence for Dcp1a was subcloned from plasmid pEGFP-DCP1a-C1 (Aizer and Shav-Tal, 2008), a gift from Dr Yaron Shav-Tal, Bar-Ilan University, Israel), into pmCherry-C1 by using XhoI and EcoRI to form pmCherry-DCP1a-C1. Transient transfections were performed using Effectene Transfection reagent (QIAGEN), according to manufacturer's instructions.

The following oligonucleotides, based on Ambion siRNA sequences available from the Sigma MISSION shRNA library, were used for lentiviral knockdown. MBNL1 shRNA targeting the 3′UTR; sense: 5′-CCGGGAGTAAAGGACGAGGTCATTACTCGAGTAATGACCTCGTCCTTTACTCTTTTTTG-3′; antisense: 5′-AATTCAAAAAAGAGTAAAGGACGAGGTCATTACTCGAGTAATGACCTCGTCCTTTACTC-3′. CUGBP1 shRNA targeting the 3′UTR: sense: 5′-CCGGCGTCAAGTACATCGTCCAAATCTCGAGATTTGGACGATGTACTTGACGTTTTTG-3′; antisense: 5′-AATTCAAAAACGTCAAGTACATCGTCCAAATCTCGAGATTTGGACGATGTACTTGACG-3′.

Oligonucleotide sequences were phosphorylated, annealed and then ligated into Tet-pLKO-puro (Addgene plasmid #21915) (Wiederschain et al., 2009), to generate plasmids pLKO_MBNL1 and pLKO_CUGBP1.

### Cell lines and cell culture

Human lens epithelial cells (HLECs) from DM1 patients (DMCat1-4) and from age-matched controls (CCat1 and CCat2) have been described previously (Rhodes et al., 2006). Cells were grown at 37°C under 5% CO_2_ in Dulbecco's modified Eagle’s medium (DMEM, Sigma) supplemented with 10% foetal bovine serum (FBS, Sigma), 1% penicillin/streptomycin (Sigma) and 1% L-glutamine (Sigma). HeLa cells were grown at 37°C under 5% CO_2_ in DMEM supplemented with 10% FBS, 1% penicillin/streptomycin. Following transduction with lentiviral vector, medium was supplemented with 250 ng/ml puromycin and 200 µg/ml G418 geneticin (G 418 disulfate salt; Sigma).

Cell lines HeLa_CTG12_GFPMBNL1 and HeLa_CTG960_GFPMBNL1 were generated in two stages. First, HeLa cells (originally obtained from ATTC) were transfected with construct pcDNA3-rtTA3 and selected for its expression by using G418 geneticin. Individual clones were generated and screened for low background expression and high induction levels of rtTA3. The best-performing clone was co-transfected with either pBI-Tet-CTG12_GFPMBNL1 or pBI-Tet-CTG960_GFPMBNL1 and a linear puromycin marker (Invitrogen), and selected with puromycin. Clonal cell lines were then screened for the formation of inducible nuclear foci following addition of 1 µg/ml of the tetracycline equivalent doxycycline (Dox), using fluorescence *in situ* hybridisation (Cy3-CAG10 probe) and fluorescence microscopy (GFPMBNL1). These cell lines were maintained under conditions identical to those used for the parental HeLa cell line, with G418 geneticin and puromycin added periodically to ensure retention of the added sequences. When required, expression of the constructs was induced using 1 µg/ml Dox for 24, 48 or 72 h as indicated.

To generate cell lines with reduced expression of MBNL1 or CUGBP1, lentiviral particles were produced using 293T cells and transduction of HeLa cells was performed as described by Wiederschain et al. (2009). Luciferase (control) plasmid was Tet-pLKO-puro_shLUC (Harwardt et al., 2016; a gift from Dr Christina Paulus, University of St Andrews). Following selection with puromycin, the mixed populations of transduced cells produced were designated lines HeLa_pLKO_MBNL1, HeLa_pLKO_CUGBP and HeLa_pLKO_Luc. All cell lines have recently been tested for contamination.

### Drug treatment

For SG formation, cells were treated with 0.25 mM NaAsO_2_ in DMEM for the times indicated before fixation, for immunocytochemistry and RNA fluorescence in situ hybridisation (FISH). For experiments that required pre-treatment with NaAsO_2_, cells were treated with 0.5 mM NaAsO_2_ for 45 min or 90 min to ensure complete formation of SGs. Recovery of SGs was initiated by washing cells several times with normal DMEM. To assess responses to 1,6-hexanediol, cells were pre-treated with NaAsO_2_ as above if required, followed by addition of 5% w/v 1,6-hexanediol to the medium for 20 min before fixation. For live-cell experiments, cells were maintained in DMEM with or without NaAsO_2_. Where possible, downstream analysis was carried out with the researcher blinded to the identity of the sample.

### Western blotting

Cells were lysed with ice-cold nuclear lysis buffer [50 mM Tris-HCl pH7.5, 0.5 M NaCl, 1% IGEPAL CA-630, 1% sodium deoxycholate, 0.1% SDS, 2 mM EDTA, cOmplete protease inhibitor cocktail (Roche)] for 5 min, then passed through a QIAshredder (QIAGEN) during 2 min centrifugation at 12,000 ***g***. Lysates were electrophoresed in a 10% acrylamide gel and transferred onto a nitrocellulose membrane (GE Healthcare) using a semi-dry blotter (BioRad). Skimmed milk powder (5%) in PBS was used as blocking solution before protein detection using primary antibodies mouse anti-MBNL1 (1:50; gift from Glenn Morris; Holt et al., 2007); rabbit anti-GFP (Abcam ab290, 1:500); rabbit anti-CUGBP1 (Abcam ab129115, 1:1000); rabbit anti-TIA1 (Proteintech 12133-2-AP; 1:500); mouse anti-tubulin (Sigma; 1:500) and secondary antibodies goat anti-rabbit-AlexaFluor790 and goat-anti-mouse-AlexaFluor680 (both Invitrogen; 1:20,000). Blots were imaged using a Licor Odyssey CLx.

### Immunocytochemistry

Cells were seeded on 18 mm square glass coverslips and incubated in normal DMEM for 24 h minimum. Drug treatments or transient transfections using Effectene Reagent (Qiagen) according to the manufacturer's instructions were carried out as required. Fixation was performed using 3.7% paraformaldehyde (PFA) (Sigma) in PHEM buffer (60 mM PIPES, 25 mM HEPES, 10 mM EGTA, 2 mM MgCl_2_ pH 6.9) for 10 min at room temperature. Immunostaining was carried out essentially as described by Sleeman et al. (2003), with 1% goat serum in PBS as blocking buffer and antibody incubation for 1 h. Primary antibodies were Mbla mouse anti-MBNL1 (Holt et al., 2007) 1:15; rabbit anti-TIA1 (Proteintech, #12133-2-AP) 1:50; rabbit anti-CUGBP1 (Abcam, #ab129115, 1:100); rabbit anti-GE1 (Cell Signalling Technology, #2548, 1:400). Secondary antibodies were goat-anti-rabbit AlexaFluor594, goat-anti-mouse AlexaFluor488, goat anti-mouse and goat-anti-rabbit Cy5 (All Jackson Laboratories, 1:250). 1.6 µg/ml DAPI (Sigma Aldrich) in H_2_O was used to stain the nucleus. Coverslips were mounted using ProLong Gold Antifade (Invitrogen).

### RNA fluorescence *in situ* hybridisation

For RNA fluorescence *in situ* hybridisation (FISH), cells were seeded on 18 mm square glass coverslips and incubated in normal DMEM or DMEM supplemented with 1 µg/ml Dox as appropriate. Cells were fixed using 3.7% PFA (Sigma) in diethylpyrocarbonate (DEPC)-treated PBS and permeabilised with 70% ethanol in DEPC-treated H_2_O for 1 h. A Cy3 dye-labelled CAG_10_ DNA probe (5′-/5Cy3/(CAG)_10_, Integrated DNA Technologies) was diluted in hybridisation buffer (2× SSC; 10% w/v dextran sulfate; 10% formamide) to a final concentration of 200 nM and incubated with the samples for 4 h at 37°C. Samples were washed with 2× SSC, 3×5 min. 1.6 µg/ml DAPI (Sigma Aldrich) in H_2_O was used to stain the nucleus. Coverslips were mounted using ProLong Gold Antifade (Invitrogen).

### Microscopy and image processing

Fixed samples were imaged using a 1.35na 100× objective on an Olympus DeltaVision RT microscope (Applied Precision) with 2×2 binning used for weaker samples and 0.2 μm sectioning for *z*-stacks. Exposure times using DAPI, FITC, TRITC and Cy5 filter sets were chosen to aim for maximum intensities of 3600. Where indicated, deconvolution was carried out by using Volocity 6.3 image analysis software (Quorum Technologies) with calculated point-spread functions. Object identification for quantitative analyses was carried out using the quantitation module in Volocity 6.3, as illustrated in Fig. S4.

### High-resolution image capture and analysis

Images were captured using the Zeiss LSM 880 laser scanning microscope using the Airyscan detector with either the ×63 or ×100 objectives. This detector increases the resolution of the final image from a theoretical maximum of 0.2 µm to 0.1 µm (Huff, 2015). Correlation parameters were collected from the images by using a custom programme written in the Volocity quantitation module (Quorum Technologies): GFPMBNL1-positive structures were identified using the Otsu method (Otsu, 1979), objects in the nucleus were removed by identifying those intersecting with DAPI-labelled regions. All remaining cytoplasmic structures were investigated for the correlation between GFPMBNL1 and CUGBP1 intensities.

### Prediction of prion-like domains

The amino acid sequences of MBNL1 and CUGBP1 were analysed using the Prion-like Amino Acid Composition (PLAAC) program (http://plaac.wi.mit.edu/; Lancaster et al., 2014). The parameters used were ‘core length: 30’ and ‘background frequency: homo sapiens’.

### Live-cell imaging

Cells were seeded onto 40 mm round coverslips (Intracel) for 24 h before transfections or transductions (where appropriate) were performed. After an additional 24 h, the coverslips were placed into a POC-R imaging chamber (Zeiss) within an environmental incubator (Solent Scientific) on an Olympus DeltaVision RT microscope (Applied Precision), with a quantifiable laser module including 488 nm and 405 nm lasers, and either maintained at 37°C with 5% CO_2_ or incubated in CO_2_-independent medium (Life Technologies). *z*-stacks of images separated by 500 nm were collected at a minimum of 3-min intervals by using a 1.35NA 100× objective. Exposure times were optimized to give a maximum intensity value of ∼1000 in each *z*-stack or in the first time-point of each time-lapse series.

### Fluorescence recovery after photobleaching

Fluorescence recovery after photobleaching (FRAP) was carried out using an Olympus DeltaVision RT microscope using a 1.35NA 100× objective, with the chamber heated to 37°C. A 488 nm laser at 100% power was used to photobleach GFP for 2.5 s, aiming to obtain ∼50% bleaching efficiency. A time-course applying the FITC filter was taken by taking three images before and 25 images after the laser fire, using adaptive time intervals as implemented in the DeltaVision software. Where appropriate, P-bodies were identified using pmCherry-Dcp1A, viewed through the TRITC filter before FRAP. Image analysis was carried out using Volocity 6.3 image analysis software (Quorum Technologies). The photobleached region was indicated by a point and the intensity value measured against time. The values were corrected for background signal and photobleaching, and normalised by setting the intensity immediately after the firing event as 0% and the highest of the pre-fire images as 100%. A one-phase association curve analysis was used in Prism 8 (GraphPad) to determine the mobile fraction and half-time of recovery for each data set.

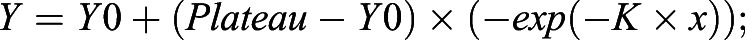
with *Y* referring to the relative fluorescence intensity, *Y*0 to the *Y* value when *x* (time) is zero, plateau to the *Y* value at infinite times, *K* to the rate constant and *x* to time.

### Statistical analyses

All statistical analyses, including ANOVA, *t*-tests, Mann–Whitney *U* test, D'Agostino–Pearson omnibus K2 normality test and removal of outliers as appropriate were carried out using Prism 8 (GraphPad). For analyses where all relevant datasets showed normal distribution, a one-way ANOVA test was used. For analyses where some or all datasets failed the D'Agostino–Pearson omnibus K2 normality test, Mann–Whitney *U* test was used. Optimal sample sizes to detect a medium (0.25) effect size at significance level <0.05 with 90% power were determined using R.

## Supplementary Material

Supplementary information
