# Supplementary material for: Condensation properties of stress granules and processing bodies are compromised in myotonic dystrophy type 1
Source: Dis Model Mech. 2022 Aug 2;15(7):dmm049294. doi: 10.1242/dmm.049294 (PMC9366894; doi:10.1242/dmm.049294)
Supplement: Supplementary information [file dmm-15-049294-s1.pdf]

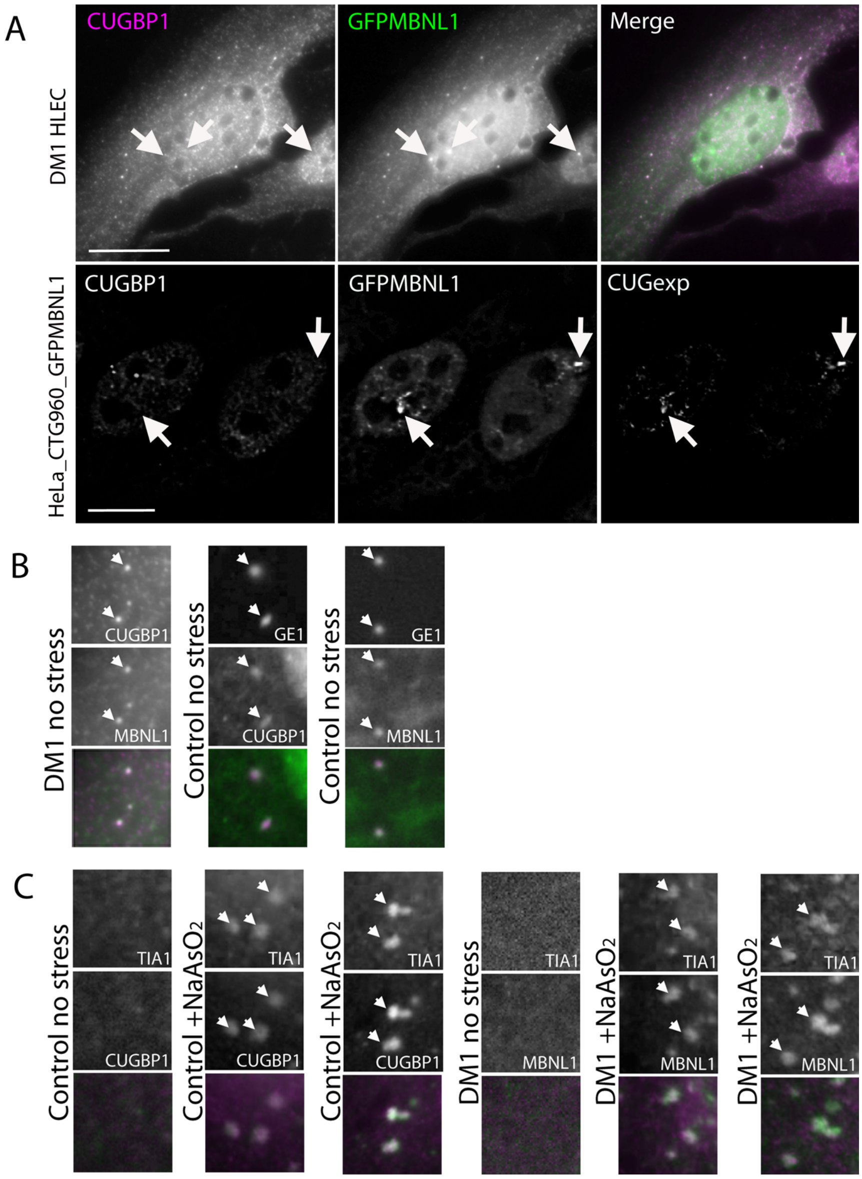

Fig. S1. (related to figures 1 and 3): CUGBP1 does not accumulate in nuclear CUGexp RNA foci. A) Human lens epithelial cells (top row) stained with antibodies against endogenous CUGBP1 (left hand panel and magenta on overlay) and MBNL1 (centre panel and green on overlay). Nuclear foci (arrows) contain MBNL1 but not CUGBP1. Cells from line HeLa_CTG960_GFPMBNL1 (bottom row) stained with antibodies against endogenous CUGBP1 (left hand panel) and with an RNA FISH probe against CUGexp RNA (right hand panel). GFPMBNL1, but not CUGBP1, accumulates in nuclear CUGexp RNA foci (arrows). Bar = 10 µm. B) CUGBP1and MBNL1 co-localise in cytoplasmic structures in unstressed HLECs (left hand panel, CUGBP1 top and magenta on overlay; MBNL1 centre and green on overlay). These structures are P-bodies, confirmed by co-localisation with GE1 (centre and right hand panels, GE1 top and magenta on overlay; CUGBP1/MBNL1 centre and green on overlays).

Disease Models & Mechanisms • Supplementary information

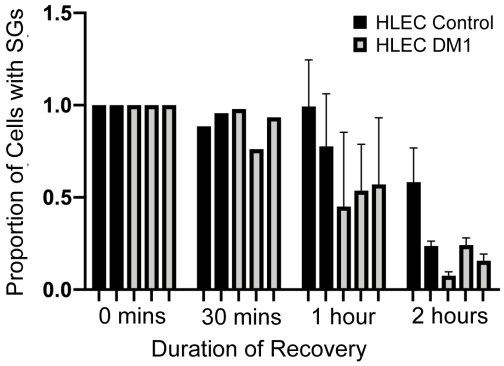

Fig. S2. (related to figure 2): Loss of Stress Granules in individual HLEC lines. Graph of percentage of cells containing stress granules in each HLEC line detected with antibodies to endogenous TIA1 and MBNL1 following recovery from sodium arsenite treatment of 30 minutes, 1hr or 2hrs, normalised to the percentage of cells in each line showing stress granules following 90 minutes of drug treatment. Black bars represent control cell lines and grey bars DM1 cell lines.

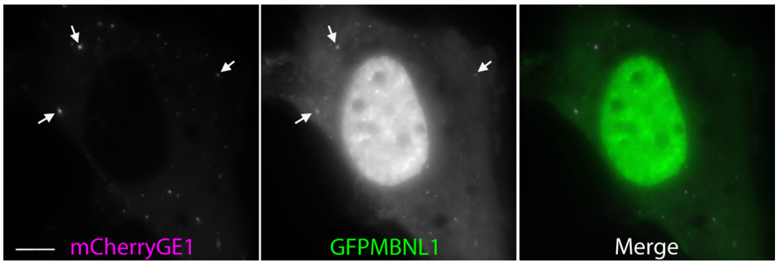

Fig. S3. (related to figure 2): Verification of PB identity in living cells prior to FRAP analysis. Human lens epithelial cells were transiently transfected with plasmids to express GFPMBNL1 (centre and green on merge) and mCherryGE1 (left and magenta on merge), a marker for P-bodies. Cytoplasmic foci conclusively identified as P-bodies using mCherryGE1 (arrows) were targeted for bleaching in FRAP experiments. Bar = 5μm.

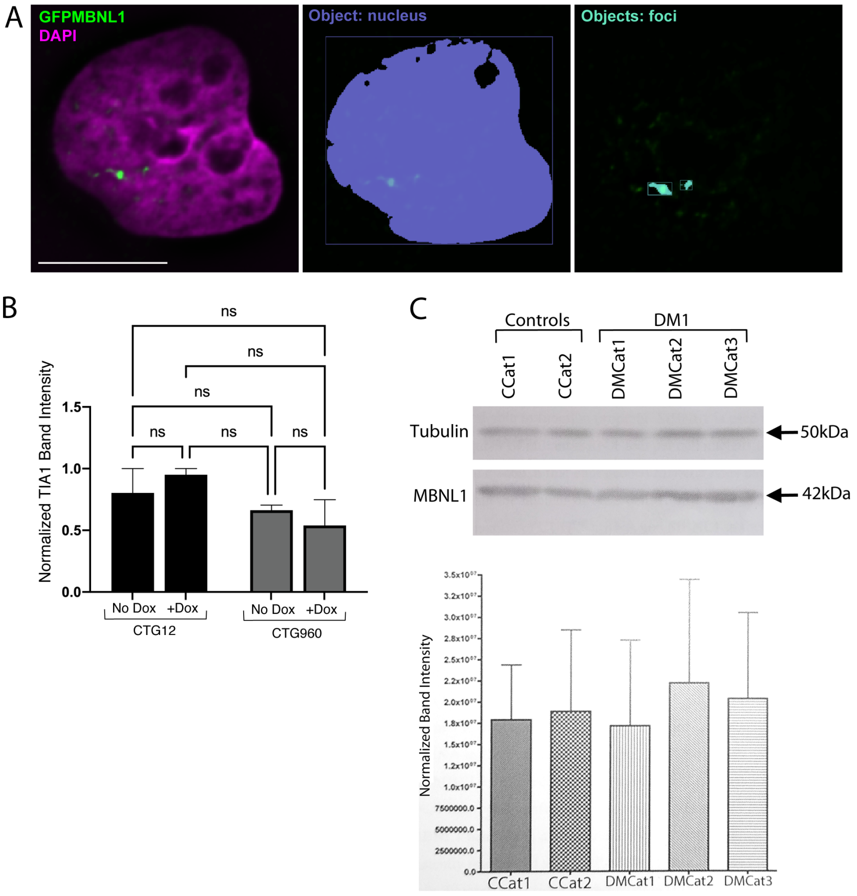

Fig. S4. (related to figure 3): Quantitation of GFPMBNL1 and MBNL1 in cell models of DM1. A) Example of object identification used to calculate the % of total nuclear GFPMBNL1 in nuclear CUGexp foci. Signals from the original images (left) were used to identify foci using GFPMBNL1 (right, cyan objects) and the nuclear volume using DAPI (centre, purple object). Bar=10μ m. B) Endogenous TIA1 shows no significant changes in expression in CTG12 and CTG960 cells with and without Doxycycline induction (ANOVA test, mean of two biological repeats adjusted for gel loading using tubulin). C) Expression of endogenous MBNL1 is not altered in HLEC lines from DM1 patients compared to controls. Representative western blot of MBNL1 and tubulin as a loading control in 3 DM1 lines and 2 control lines with quantitative analysis from 3 independent experiments.

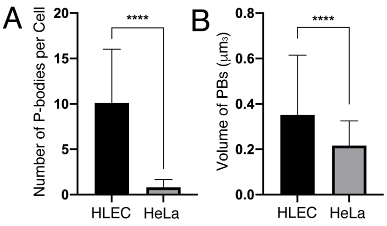

Fig. S5. (related to Figure 4): Differences in PB Characteristics in HLEC compared to HeLa Cells and between DM1 and control HeLa cell lines. A) Numbers of PBs per cell are lower in HeLa cells compared to HLECs. B) Volumes of PBs are smaller in HeLa cells compared to HLECs (n=514 cells HLEC pooled from control and DM1 lines from 2 independent experiments, n=287 cells HeLa pooled from CTG12 and CTG960 lines from 2 independent experiments, Mann-Whitney test, P<0.0001).

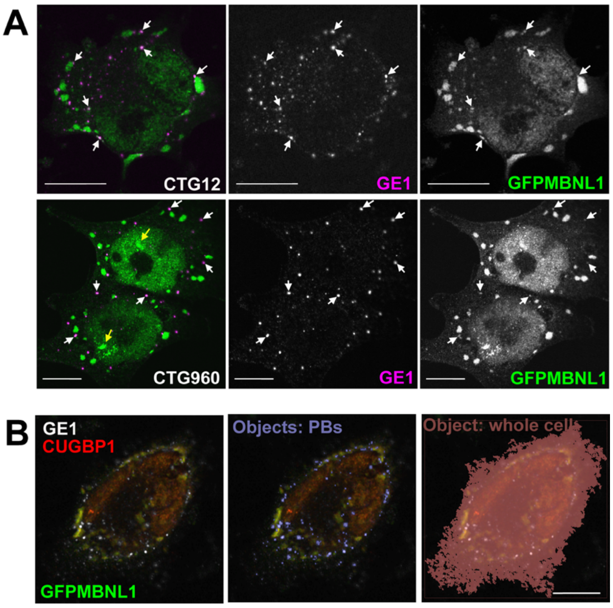

Fig. S6. (related to figure 4): Quantitation of GFPMBNL1 in P-bodies in HeLa cell model of DM1. A) Cells from line CTG12 (top) and CTG960 (bottom) treated with sodium arsenite contain large numbers of P-bodies detected with antibodies against GE1 (centre and magenta on merge) in addition to Stress granules. Some, but not all, of these P-bodies contain detectable amounts of GFPMBNL1 (right and green on merge, approx 39% in CTG12 and 38% in CTG960), examples marked by white arrows. Yellow arrows denote CUGexp nuclear foci containing GFPMBNL1. B) Example of object identification used to calculate the % of total cellular GFPMBNL1 per PB. Signals from the original images (left) were used to identify P-bodies using GE1 (centre, purple objects) and the entire cell volume using CUGBP1 (right, brown object). Bar=10μm.

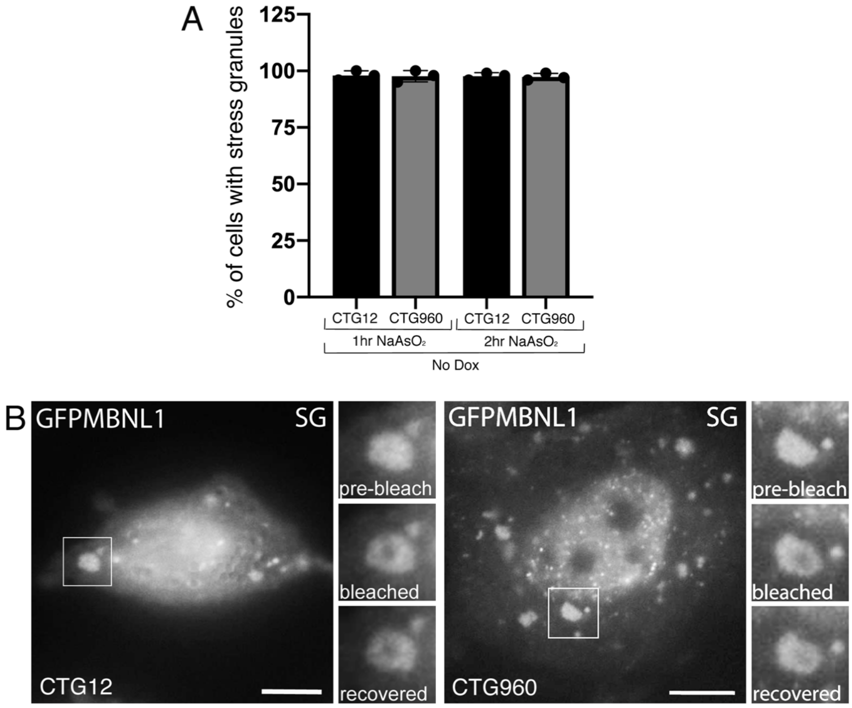

Fig. S7. (related to Figure 5): Stress Granule dynamics in HeLa cell model of DM1. A) In CTG12 and CTG960 cell lines without doxycycline induction of the DMPK mini-gene and GFPMBNL1, no differences are seen between the percentage of cells containing stress granules detected with endogenous TIA1 and MBNL1 after 1hr or 2hrs treatment with sodium arsenite (3 independent experiments, n=50 cells per cell line per time point per experiment). B) Examples of bleaching within stress granules containing GFPMBNL1 used for FRAP analysis. Large panels show the whole cell pre-bleach. Smaller panels show the bleached SG before bleaching, after bleaching and following recovery. Bar = 10µm.

Disease Models & Mechanisms • Supplementary information

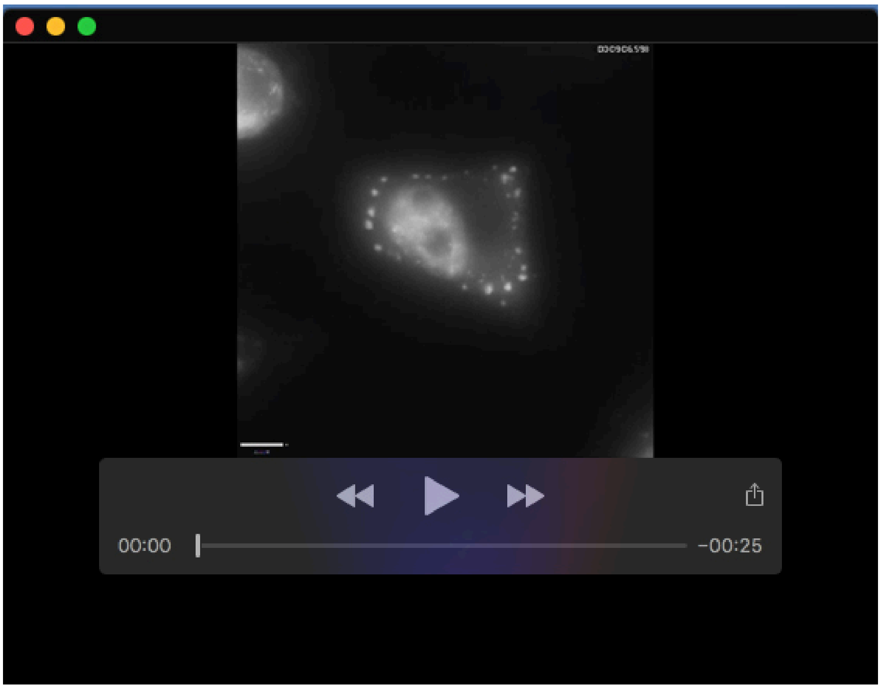

Movie 1. SG formation (CTG12) Maximum intensity z-projection of time-lapse movies showing the formation of cytoplasmic stress granules in cells from line CTG12 treated with sodium arsenite (related to figure 5).

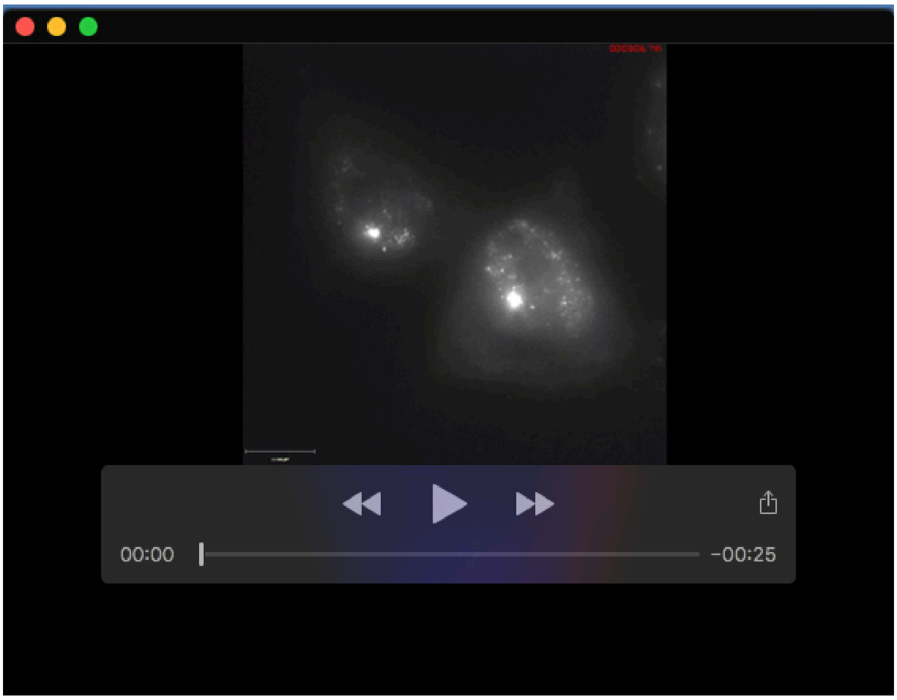

Movie 2. SG formation (CTG960) Maximum intensity z-projection of time-lapse movies showing the formation of cytoplasmic stress granules in cells from line CTG960 treated with sodium arsenite (related to figure 5).

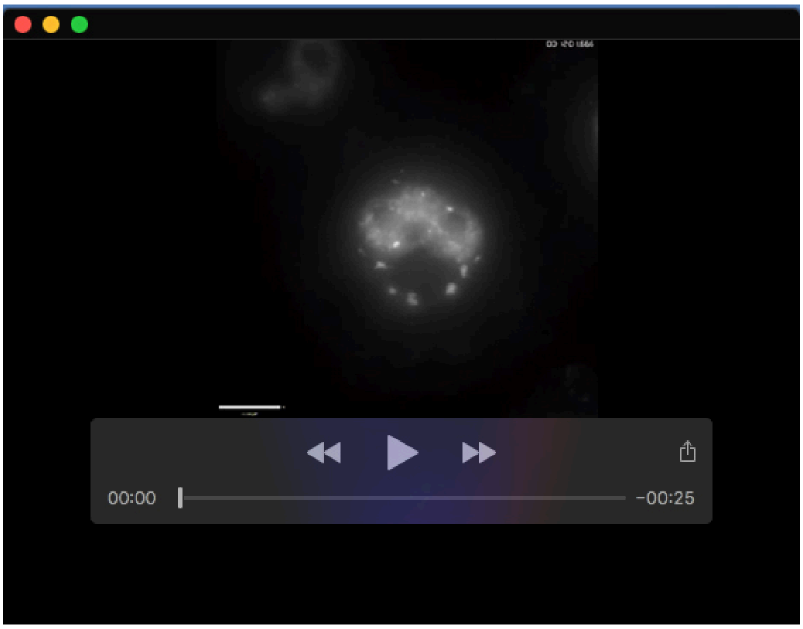

Movie 3. SG dispersal Maximum intensity z-projection of time-lapse movies showing the loss of cytoplasmic stress granules from cells from line CTG12 following sodium arsenite removal. The splitting of Stress granules into smaller structures prior to their complete loss can be seen. Time indicated is relative to the start of imaging, rather than the initiation of recovery from stress (related to figure 5).

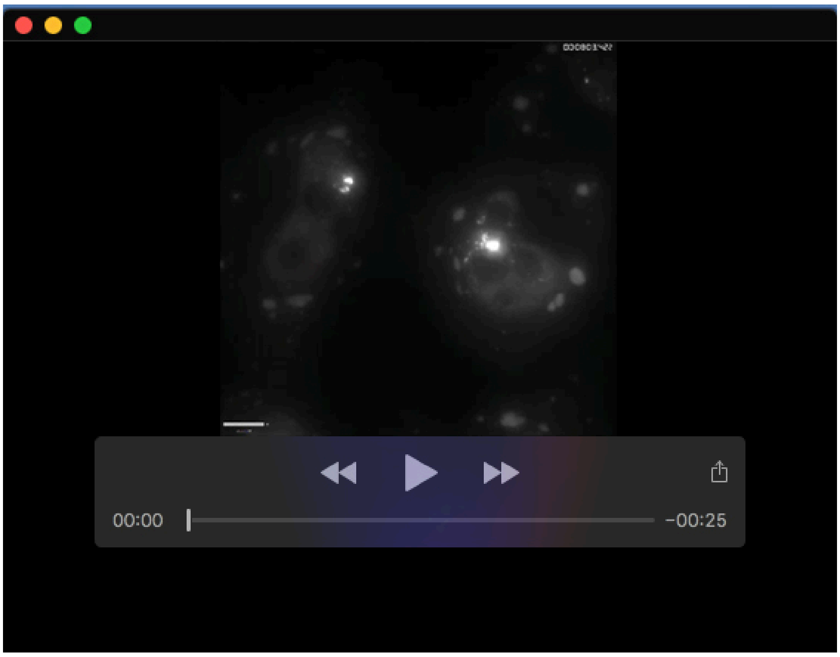

Movie 4. SG dispersal Maximum intensity z-projection of time-lapse movies showing the loss of cytoplasmic stress granules from cells from line CTG960 following sodium arsenite removal. The splitting of Stress granules into smaller structures prior to their complete loss can be seen. Time indicated is relative to the start of imaging, rather than the initiation of recovery from stress (related to figure 5).

Disease Models & Mechanisms • Supplementary information
